# Pan-cancer experimental characteristic of human transcriptional patterns connected with telomerase reverse transcriptase (*TERT*) gene expression status

**DOI:** 10.3389/fgene.2024.1401100

**Published:** 2024-05-27

**Authors:** Aleksey Drobyshev, Alexander Modestov, Maria Suntsova, Elena Poddubskaya, Alexander Seryakov, Aleksey Moisseev, Maksim Sorokin, Victor Tkachev, Galina Zakharova, Aleksander Simonov, Marianna A. Zolotovskaia, Anton Buzdin

**Affiliations:** ^1^ Endocrinology Research Center, Moscow, Russia; ^2^ Institute of Personalized Oncology, I. M. Sechenov First Moscow State Medical University, Moscow, Russia; ^3^ Clinical Center Vitamed, Moscow, Russia; ^4^ Medical Holding SM-Clinic, Moscow, Russia; ^5^ Oncobox LLC, Moscow, Russia; ^6^ Moscow Center for Advanced Studies 20, Moscow, Russia; ^7^ Shemyakin-Ovchinnikov Institute of Bioorganic Chemistry, Moscow, Russia

**Keywords:** TERT promoter C228T and C250T mutations, reverse transcriptase, differential gene expression analysis, RNA sequencing, pathway activation profiling, pan-cancer investigation

## Abstract

The *TERT* gene encodes the reverse transcriptase subunit of telomerase and is normally transcriptionally suppressed in differentiated human cells but reactivated in cancers where its expression is frequently associated with poor survival prognosis. Here we experimentally assessed the RNA sequencing expression patterns associated with *TERT* transcription in 1039 human cancer samples of 27 tumor types. We observed a bimodal distribution of *TERT* expression where ∼27% of cancer samples did not express *TERT* and the rest showed a bell-shaped distribution. Expression of *TERT* strongly correlated with 1443 human genes including 103 encoding transcriptional factor proteins. Comparison of *TERT*- positive and negative cancers showed the differential activation of 496 genes and 1975 molecular pathways. Therein, 32/38 (84%) of DNA repair pathways were hyperactivated in *TERT+* cancers which was also connected with accelerated replication, transcription, translation, and cell cycle progression. In contrast, the level of 40 positive cell cycle regulator proteins and a set of epithelial-to-mesenchymal transition pathways was specific for the *TERT- *group suggesting different proliferation strategies for both groups of cancer. Our pilot study showed that the *TERT+* group had ∼13% of cancers with *C228T* or *C250T* mutated *TERT* promoter. However, the presence of promoter mutations was not associated with greater *TERT* expression compared with other *TERT+* cancers, suggesting parallel mechanisms of its transcriptional activation in cancers. In addition, we detected a decreased expression of L1 retrotransposons in the *TERT+* group, and further decreased L1 expression in promoter mutated *TERT+* cancers. *TERT* expression was correlated with 17 genes encoding molecular targets of cancer therapeutics and may relate to differential survival patterns of *TERT*- positive and negative cancers.

## 1 Introduction

The human telomerase reverse transcriptase gene *TERT* encodes a reverse transcriptase that maintains chromosome ends by adding hexameric repeats 5′-*TTAGGG*-3′ to the 3′termini using an RNA template encoded by the *TERC* gene (Wu et al., 2017). In the human body, telomerase activity could be found in few types of the somatic cells, such as activated B-lymphocytes and T-lymphocytes, endothelial cells and macrophages (Hsiao et al., 1997; Weng et al., 1998; Lin et al., 2010; Gizard et al., 2011). In general, telomerase expression is normally restricted to developing tissues, germ-line and stem cells but it can be also pathologically reactivated in cancers, thus supporting continuous proliferation and preventing senescence caused by shortened telomeres (Colebatch et al., 2019). Furthermore, expression of telomerase was reported as a prognostic factor of patient survival in some cancers, where an increased *TERT* expression is a negative prognostic biomarker (Gong et al., 2021; Kwon and Yi, 2021; Li et al., 2024). Thus, the analysis of expression patterns and structural changes of the *TERT* gene is of great interest. The increased expression of *TERT* was frequently reported to be associated with activating somatic mutations in its promoter region (e.g., (Huang et al., 2015)). However, the frequency of such promoter mutations differs significantly by cancer type (Blackburn et al., 2015). The highest frequency of *TERT* promoter mutations was found in melanomas and central nervous system tumors (in particular, over 70% in primary glioblastoma and oligodendroglioma cases) (Killela et al., 2013; Simon et al., 2015; Hugdahl et al., 2018; Colebatch et al., 2019; Olympios et al., 2021; Guo et al., 2022). *TERT* promoter mutations are also frequently observed in urothelial, thyroid, and hepatocellular carcinomas, and in squamous cell and basal cell carcinoma of the skin (Griewank et al., 2013; Scott et al., 2014; Liu and Xing, 2016; Nault and Zucman-Rossi, 2016; Colebatch et al., 2019; Hayashi et al., 2021; Zhang et al., 2022). In addition, mutations were found in most tumor types, but at a relatively low frequency (Vinagre et al., 2013; Bell et al., 2016; Gupta et al., 2021; El Zarif et al., 2023).

The significance of *TERT* promoter mutations in carcinogenesis has been assessed in many studies (Fredriksson et al., 2014; Barthel et al., 2017). It was hypothesized that it mainly occurs in tumors originating from tissues with a low self-renewal rate (Killela et al., 2013; Simon et al., 2015).

Two activating mutations in the promoter region of *TERT*: *C228T* (chr5:1,295,228 C>T on assembly GRCh37; NM_198253.3:c.-124C>T) and *C250T* (chr5:1,295,250 C>T on assembly GRCh37; NM_198253.3:c.-146C>T) are located at the positions −124 and −146 bp, respectively, upstream of the ATG site and are the most common reported mutations in the *TERT* gene (Colebatch et al., 2018; Powter et al., 2021; Kang et al., 2022). These mutations are typically heterozygous, rarely occur simultaneously and result in a 5′-*CCCGGAAGGGG*-3′ sequence of 11 bases that corresponds to a *de novo* binding site for the E26 transformation-specific transcriptional factor (ETS) protein (da Costa et al., 2021; Hasanau et al., 2022; Koleilat et al., 2023). In addition, *TERT* promoter mutations are associated with the switching of inactive chromatin marks to active ones in the *TERT* promoter (Stern et al., 2015; Salgado et al., 2020). It increases the transcription and thereby leads to enhanced activity of telomerase (Liu et al., 2016). It was also previously reported that in humans *TERT* is frequently expressed in the form of alternatively spliced transcripts in both normal and tumor cells, and only the longest transcripts possess the reverse transcriptase enzymatic activity (Hrdličková et al., 2012; Colebatch et al., 2019). Overexpression of some alternatively spliced variants could increase the proliferation rate without enhancing telomerase activity (Hrdličková et al., 2012).

In a number of studies, mutations in the promoter region of *TERT* were reported to correlate with its increased expression level. In melanomas, the increased telomerase expression was correlated with *C228T/A* and *C250T/A TERT* promoter mutations (Lee et al., 2016; Shaughnessy et al., 2020; Guo et al., 2022). Also, higher expression of *TERT* in tumors with promoter mutations was observed in urothelial carcinomas and in gliomas (Borah et al., 2015; Heidenreich et al., 2015).

However, these findings are contradictory as some researchers reported lack of statistical significance for such a phenomenon for patients diagnosed with thyroid cancer (Pestana et al., 2020), bladder cancer (Allory et al., 2014), and in melanoma cell lines (Salgado et al., 2020). Also, it was reported that the *TERT* promoter mutation status did not affect its mRNA level in hepatocellular carcinomas (Chen et al., 2014; Zhang et al., 2022). A recent pan-cancer study reported only a marginally significant association between promoter mutation status of *TERT* and its mRNA level (*p* = 0.0373, N = 503 cell lines) (Stern et al., 2020). These facts could be attributed to the structural variations of the *TERT* gene. For example, it was found that nearly 50% of glioblastoma patients with wild-type *TERT* promoter harbored structural rearrangements of *TERT* (Diplas et al., 2018). At the same time there was no significant difference in *TERT* expression between the rearranged and promoter-mutant glioblastoma subgroups. Moreover, it was reported that telomerase-expressing embryonic stem cells with an introduction of any of the most frequent *TERT* promoter mutations showed only a modest increase in this gene transcription with no detectable impact on telomerase functional activity (Chiba et al., 2015).

Interestingly, recent studies have identified several non-canonical functions of *TERT* that are not related to maintaining telomere stability (Ségal-Bendirdjian and Geli, 2019; Thompson and Wong, 2020; Akincilar et al., 2021). For example, *TERT* can act as a modulator in NF-kB and Wnt/β-catenin signaling pathways (Li and Tergaonkar, 2014). Other non-canonical functions of *TERT* include regulation of apoptosis, maintenance of the cellular redox homeostasis, cell proliferation, response to DNA damage, and formation of G-quadruplexes in the G-rich area of gene promoters, which can influence transcription by regulating binding of transcription factor proteins (Fleisig et al., 2016; Khattar et al., 2016; Yuan et al., 2019; Rosen et al., 2020; Gu et al., 2022; Pavlova et al., 2022; Palamarchuk et al., 2023). Thus, theoretically *TERT* promoter mutations may also affect these and probably other processes related to *TERT*.

In this study, we experimentally assessed the gene expression patterns associated with the status of *TERT* expression in 1039 human cancer samples of 27 tumor types investigated by RNA sequencing. On pan-cancer level we showed a bimodal distribution of *TERT* expression where ∼27% of cancer samples did not express *TERT*, whereas the rest showed a bell-shaped distribution. Expression of *TERT* was strongly correlated with 1443 human genes including 103 encoding transcriptional factor proteins. Comparison of *TERT*- positive and negative cancers showed the differential activation of 496 genes and 1975 molecular pathways. Among them, 32/38 (84%) of DNA repair pathways were hyperactivated in *TERT+* cancers which was also connected with accelerated replication, transcription, translation, and cell cycle progression. Our pilot study showed that the *TERT+* group included ∼13% of cancers with mutated *TERT* promoter (mutations *C228T*, *C250T*, or both). However, the presence of promoter mutations was not associated with a greater level of *TERT* expression compared with other *TERT*+ cancers. This evidences parallel mechanisms leading to the *TERT* gene transcriptional activation with only a rather minor impact related to promoter mutation.

## 2 Materials and methods

### 2.1 Ethics

The consent procedure and the design of the study were approved by the ethical committee of Clinical Center Vitamed, Moscow, protocol date 16.10.2017.

### 2.2 Patient enrollment and biomaterials

We enrolled 1039 adult 18–80 years old male and female patients with diagnosed tumors who were either eligible for Oncobox clinical trials NCT03521245 (Sorokin et al., 2020) and NCT03724097, or submitted their biomaterials for Oncobox molecular testing. For solid tumors the biomaterials were formalin-fixed paraffin embedded (FFPE) samples of surgically removed tumor tissue or core-needle biopsies. The materials were confirmed by a certified pathologist for diagnosis and content of cancer cells (no less than 50% of cancer cells in a biosample was acceptable). All the patients or their legal representatives signed written informed consents to participate in this study and to publish the results of RNA sequencing analysis without disclosure of personal genetic data.

### 2.3 RNA sequencing and bioinformatic analysis

Total RNA preps extracted from the tumor biomaterial were subjected to RNA sequencing as previously described (Suntsova et al., 2019). Solid tissue biosamples were either immediately stabilized in RNAlater (Qiagen, Germany) and then stored at −70 C or fixed in formalin and embedded in paraffin blocks. RNA extraction was performed immediately before the preparation of sequencing libraries using QIAGEN RNeasy Kit (Qiagen) or Direct-zol™ RNA MiniPrep (Zymo Research) with TRI Reagent (MRC) for tissues in RNAlater and the RecoverAll™ RNeasy FFPE Kit (Qiagen), following the manufacturer’s protocol. Also, KAPA RNA Hyper with RiboErase (KAPA Biosystem) Kit was used for further depletion of ribosomal RNA and library preparation (Suntsova et al., 2019).

RNAseq FASTQ files were processed with STAR aligner in “GeneCounts” mode with the Ensembl human transcriptome annotation (Dobin et al., 2013). Ensembl gene IDs were converted to HUGO Gene Nomenclature Committee (HGNC) gene symbols using the Complete HGNC dataset (https://www.genenames.org/, database version from 13 July 2017). Overall, expression levels were established for 35,126 annotated genes with the corresponding HGNC identifiers. For further assessments, only the samples with high-quality experimental RNA sequencing profiles reaching the threshold of 3.5*10^6 gene-mapped reads were selected (Suntsova et al., 2019), statistics shown on Table 1. By comparing the tumor and normal expression profiles, the case-to-normal ratios for the expression of individual genes were calculated, as well as the extent of differential activation of 2929 intracellular molecular pathways (Borisov et al., 2020) using OncoboxPD tool (Zolotovskaia et al., 2022). For these comparisons, the RNA sequencing profiles of healthy tissues previously obtained by us for human donors killed in road accidents using the same protocols, equipment, and reagents were used as the normal controls (Sorokin et al., 2023).

**TABLE 1:** Statistics for experimental cancer samples used in this study sorted according to cancer type diagnoses.

Tumor type	*TERT+*	*TERT-*	*TERT* mutation status (no data)	*TERTmut* (*C250T/A* mutated)	*TERTmut* (*C228T/A* mutated)	*TERTwt*	Number of samples
Acute lymphoblastic leukemia	44 (100%)	0 (0%)	44	ND	ND	ND	44
Acute myeloid leukemia	14 (93.3%)	1 (6.7%)	15	ND	ND	ND	15
Adrenocortical carcinoma	1 (25%)	3 (75%)	4	ND	ND	ND	4
Breast cancer	80 (70.8%)	33 (29.2%)	101	-	-	12	113
Cervical cancer	19 (79.2%)	5 (20.8%)	24	ND	ND	ND	24
Cholangiocarcinoma	10 (83.3%)	2 (16.7%)	8	-	-	4	12
Colorectal cancer	131 (86.8%)	20 (13.2%)	133	-	-	18	151
Esophageal carcinoma	4 (100%)	0 (0%)	3	-	-	1	4
Glioblastoma	44 (84.6%)	8 (15.4%)	43	2	3	4	52
Head and neck cancer	9 (90%)	1 (10%)	3	-	-	7	10
Leiomyosarcoma	2 (28.6%)	5 (71.4%)	6	-	-	1	7
Lung cancer	111 (81%)	26 (19%)	129	-	-	8	137
Melanoma	16 (72.7%)	6 (27.3%)	16	1	2	3	22
Multiple myeloma	50 (92.6%)	4 (7.4%)	54	ND	ND	ND	54
Other CNS tumors	10 (28.6%)	25 (71.4%)	33	-	-	2	35
Ovarian cancer	44 (65.7%)	23 (34.3%)	61	-	-	6	67
Pancreatic adenocarcinoma	30 (71.4%)	12 (28.6%)	35	-	-	7	42
Prostate adenocarcinoma	9 (75%)	3 (25%)	10	-	-	2	12
Renal cell carcinoma	15 (48.4%)	16 (51.6%)	30	-	-	1	31
Sarcoma	7 (87.5%)	1 (12.5%)	2	-	-	6	8
Skin carcinoma	5 (100%)	0 (0%)	3	-	2	-	5
Soft tissue sarcoma	5 (33.3%)	10 (66.7%)	13	-	-	2	15
Stomach adenocarcinoma	48 (90.6%)	5 (9.4%)	45	-	-	8	53
Thymoma	3 (100%)	0 (0%)	1	-	-	2	3
Thyroid cancer	40 (38.5%)	64 (61.5%)	101	1	-	2	104
Urothelial carcinoma	4 (66.7%)	2 (33.3%)	2	-	2	2	6
Uterine corpus endometrial carcinoma	6 (66.7%)	3 (33.3%)	6	-	-	3	9
Total	761 (73.2%)	278 (26.8%)	925	4	9	101	1039

Pathway activation level (PAL) is an integral quantitative and qualitative characteristic of changes in the expression levels for genes participating in a molecular pathway (Aliper et al., 2017; Buzdin et al., 2018; Borisov et al., 2020; Zolotovskaia et al., 2023).

PAL values were calculated as follows:

$${\text{PAL}}_{p}=100*\sum_{n}{\text{ARR}}_{np}*\mathrm{lg}\;\left({\text{CNR}}_{n}\right)/\sum_{n}\left.{\left|\text{ARR}\right.}_{np}\right|,$$

where PAL*p* is PAL for a pathway *p*, CNR*n* is the case-to-normal ratio for a gene *n*; ARR (activator/repressor role) is a Boolean value that depends on the function of this gene product in the pathway *p* (Zolotovskaia et al., 2023). ARR*np* is a Boolean value defined as follows: −1 when the product of the gene *n* inhibits the pathway *p*; 1 when the product of *n* activates *p*; 0 when the product of *n* has an ambiguous role in *p*. The CNR*n* value is calculated as the ratio of a quantitative metric level for the gene *n* in a biosample under study to an average level for *n* in the control group. For each tumor type, we normalized the expression data using the group of 4-6 control biosamples of the corresponding healthy tissues obtained for either healthy donors (blood) or donors killed in road accidents (solid tissues) from the ANTE collection of RNA sequencing samples that was previously published by our group (Sorokin et al., 2023).

Principal component analysis was performed for the groups of biosamples stratified depending on the *TERT* expression (*TERT+*/*TERT-*) and *TERT* promoter mutation status (*TERTwt*/*TERTmut*).

Spearman correlation coefficients with *p-values* were calculated using the R “stats” package and visualized using the R “corrplot” package. We used a Benjamini-Hochberg adjusted *p-value* threshold 0.05 for the assessment of correlation significance.

Gene ontology (GO) enriched terms were defined for differential gene sets investigated using “clusterProfiler” R package, and *p-values* were adjusted according to Benjamini and Hochberg false discovery rate (FDR) correction procedure with the statistical threshold of 0.05. L1 retroelement quantification was calculated using the TEcount tool based on precomputed STAR alignments according to the recommendation of TEcount. Expression data of genes and L1 retroelements (of L1HS and L1PA2 subgroups) were then DESeq2 normalized. Comparisons of PAL for molecular pathways, gene expression, and L1 expression for the different groups of interest were performed using non-parametric Mann Whitney U-test in R (Benjamini-Hochberg adjusted *p-value* threshold 0.05). DESeq2 software was used to obtain the differentially expressed genes from raw counts. Genes with adjusted *p-values* < 0.05 and absolute value of fold change (FC) > 2 were considered as differentially expressed.

### 2.4 *TERT* promoter mutation assessment

In this study we investigated *TERT* promoter mutation status for 114 independent FFPE solid cancer tissue samples representing 22 tumor types: Breast cancer (12 samples), Cholangiocarcinoma (4 samples), Colorectal cancer (18 samples), Esophageal carcinoma (1 sample), Glioblastoma (9 samples), Head and neck cancer (7 samples), Leiomyosarcoma (1 sample), Lung cancer (8 samples), Melanoma (6 samples), Other CNS tumors (2 samples), Ovarian cancer (6 samples), Pancreatic adenocarcinoma (7 samples), Prostate adenocarcinoma (2 samples), Renal cell carcinoma (1 sample), Sarcoma (6 samples), Skin carcinoma (2 samples), Soft tissue sarcoma (2 samples), Stomach adenocarcinoma (8 samples), Thymoma (2 samples), Thyroid cancer (3 samples), Urothelial carcinoma (4 samples), and Uterine corpus endometrial carcinoma (3 samples).

The *TERT* promoter genomic region potentially containing *C250T/A* and *C228T/A* mutations was amplified as described before (Colebatch et al., 2018) with minor modifications. The 30-µL reaction mixture contained 5 ng of tumor-isolated genomic DNA, 500 nM of each primer (forward: 5′-*GCA​GCG​CTG​CCT​GAA​ACT​CG-*3′, reverse: 5′-*CGT​CCT​GCC​CCT​TCA​CCT​TC-*3′), 200 nM of 7-deaza-dGTP and 1x PCR mastermix (HS-Sybr, Evrogen, Russia). Thermocycling conditions were as follows: 95°C for 5 min, 4 cycles of 98°C for 14 s, 66°C for 10 s, 75°C for 30 s, followed by 40 cycles of 95°C for 10 s, 66°C for 5 s, 75°C for 14 s, followed by melting curve recording from 95°C to 75°C with 0,1°C/s rate. Samples with clear melting peak at 88°C were used for further purification. The 60 µL of PCR product were purified with MDX041 NGS Clean and Select Beads (Meridian Bioscience, United States) according to manufacturer’s protocol and eluted with 10 µL of 10 mM Tris-HCl pH 8.0; 5 µL of purified product was sequenced with 3500xL Applied Biosystems sequencer using reverse primer.

In the *TERT* promoter mutation assessment process, the *C228A* mutation (chr5:1,295,228 C>A on assembly GRCh37; NM_198253.3:c.-124C>A) was detected in one biosample of the *TERT+* group (Urothelial carcinoma), which creates a putative ETS motif (Spiegl-Kreinecker et al., 2018; Gutierrez-Rodrigues et al., 2019; Yu et al., 2021). The *C250A* mutation (chr5:1,295,250 C>A on assembly GRCh37; NM_198253.3:c.-146C>A) is also possible (Spiegl-Kreinecker et al., 2018; Gutierrez-Rodrigues et al., 2019; Yu et al., 2021) but was not detected in our study. For further analysis, *C250T/A* and *C228T/A* mutations were considered as the driver hotspot clinically relevant *TERT* promoter mutations.

### 2.5 Survival analysis

Overall survival (OS) and progression-free survival (PFS) analysis and hazard ratio calculation were assessed with relation to extent of *TERT* expression for colorectal cancer (TCGA-COAD) and thyroid cancer (TCGA-THCA) patients using the TCGA database. Patient survival analysis and plotting were performed using R packages “survival,” “survminer,” “pheatmap,” and “ggplot2”. The statistical significance of differences was measured by log-rank test *p-value*. *p-values* were adjusted according to Benjamini and Hochberg false discovery rate (FDR) correction procedure. The statistical threshold for FDR-adjusted *p-value* was 0.05. The Kaplan–Meier plots were drawn to visualize survival differences.

## 3 Results

In this study we aimed to identify transcriptomic patterns associated with cancer expression of the *TERT* gene in a high-throughput experimental assay. To this end, using the laboratory protocol and bioinformatic pipeline from (Suntsova et al., 2019), we obtained bulk RNA sequencing profiles for 1039 human cancer tissue biosamples representing 27 tumor types (Table 1). Cancer tissue specimens were obtained from the patients who were either included in clinical trials NCT03521245 (Sorokin et al., 2020) and NCT03724097, or who submitted their biomaterials for Oncobox molecular testing in 2018–2021 and agreed to participate in this study. Among them, for 114 patients with available biosamples and who gave their additional informed consent, the statuses of the *TERT* gene mutations at *C228T* and *C250T* sites were established later by Sanger sequencing. The experimental cancer RNA profiles were then bioinformatically assessed depending on the status of (i) *TERT* expression and, where available, (ii) *TERT* promoter mutation.

### 3.1 Transcriptional patterns of *TERT* expression in human cancers

**FIGURE 1:**
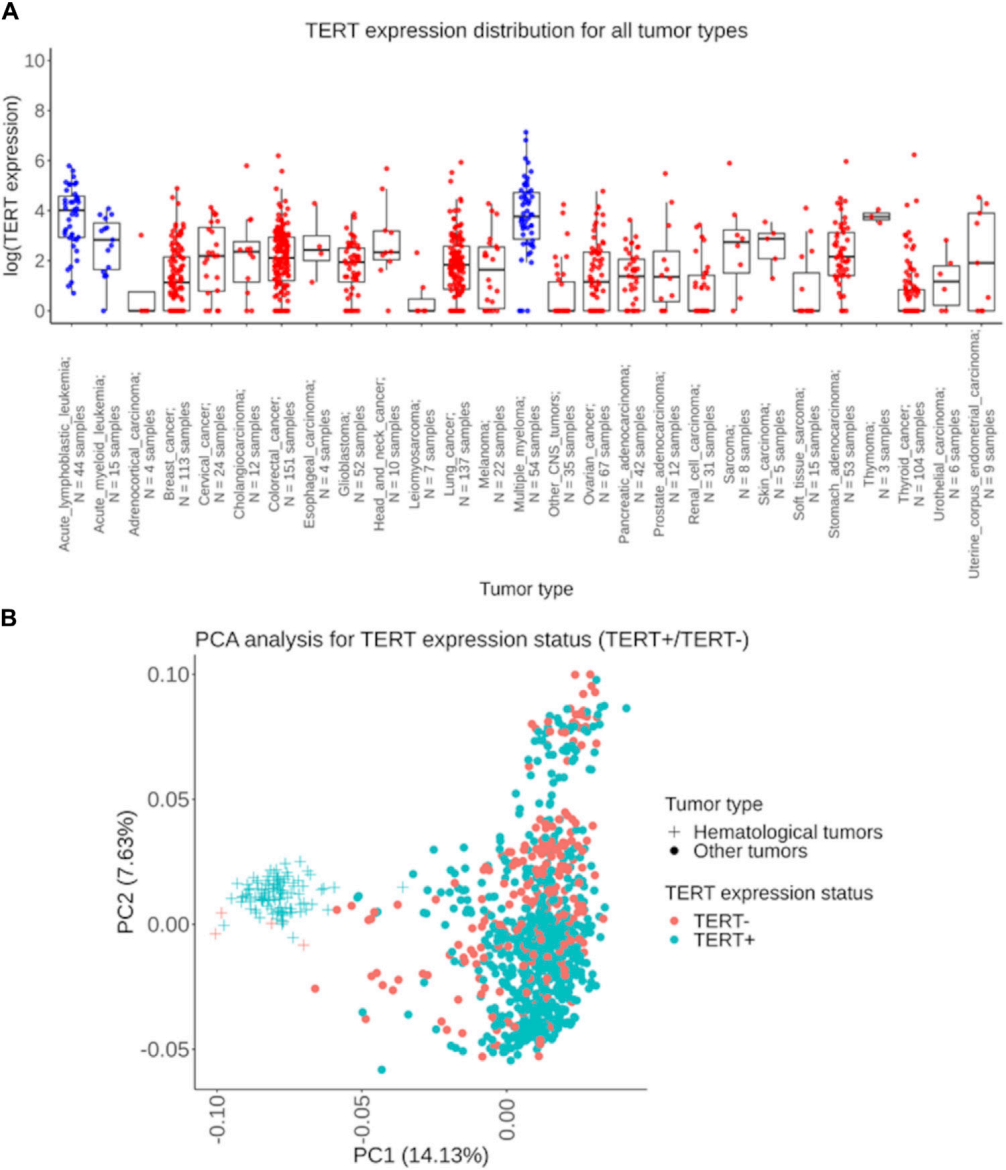
Assessment of *TERT* expression distribution in experimentally tested 1039 tumor samples. **(A)** The extent of *TERT* expression shown separately for 27 cancer types under analysis. The *Y*-axis indicates the natural logarithm of (*TERT* expression +1) value. Blue dots represent biosamples of the hematological cancers: acute lymphoblastic leukemia, acute myeloid leukemia, and multiple myeloma; red dots indicate biosamples of the non-hematological tumors. **(B)** Principal component analysis of *TERT+* and *TERT- *tumors, hematologic and solid cancers are shown separately. Dots and crosses represent specific biosamples.

Our pan-cancer analysis clearly revealed a bimodal distribution of the RNA sequencing profiles sorted according to the expression of *TERT* (Figure 1A), where ∼73% of the samples expressed *TERT*, and ∼27% showed no detectable transcription (Table 1). We detected *TERT* expression—positive (*TERT+*) status in the majority of the samples in 21 (78%) tumor types. The biggest proportion of *TERT+* samples was observed for the hematological cancers: in acute lymphoblastic leukemia (100%; *n* = 44), acute myeloid leukemia (93%; *n* = 15), and multiple myeloma (93%, *n* = 54), Table 1. Principal component analysis performed for all available expression profiles demonstrated that for most cancers, *TERT* expression status did not affect sample clustering. At the same time, a separate cluster was observed for a group of hematologic tumor samples, which were mostly *TERT+* (Figure 1B).

However, in six tumor types the proportion of *TERT* expression–negative (*TERT*-) samples was predominant: in adrenocortical carcinoma (75%; *n* = 4), in non-glioblastoma CNS tumors (71%; *n* = 35), in soft tissue sarcomas (67%; *n* = 15), in renal cell carcinoma (52%; *n* = 31), in leiomyosarcoma (71%; *n* = 7), and in thyroid cancer (62%; *n* = 104), Table 1. In 23 cancer types collectively representing 95% of the RNAseq profiles, both *TERT*+ and *TERT*- biosamples were detected, thus evidencing that the phenomenon of *TERT*- negative cancers is not restricted to specific tumor localizations.

**FIGURE 2:**
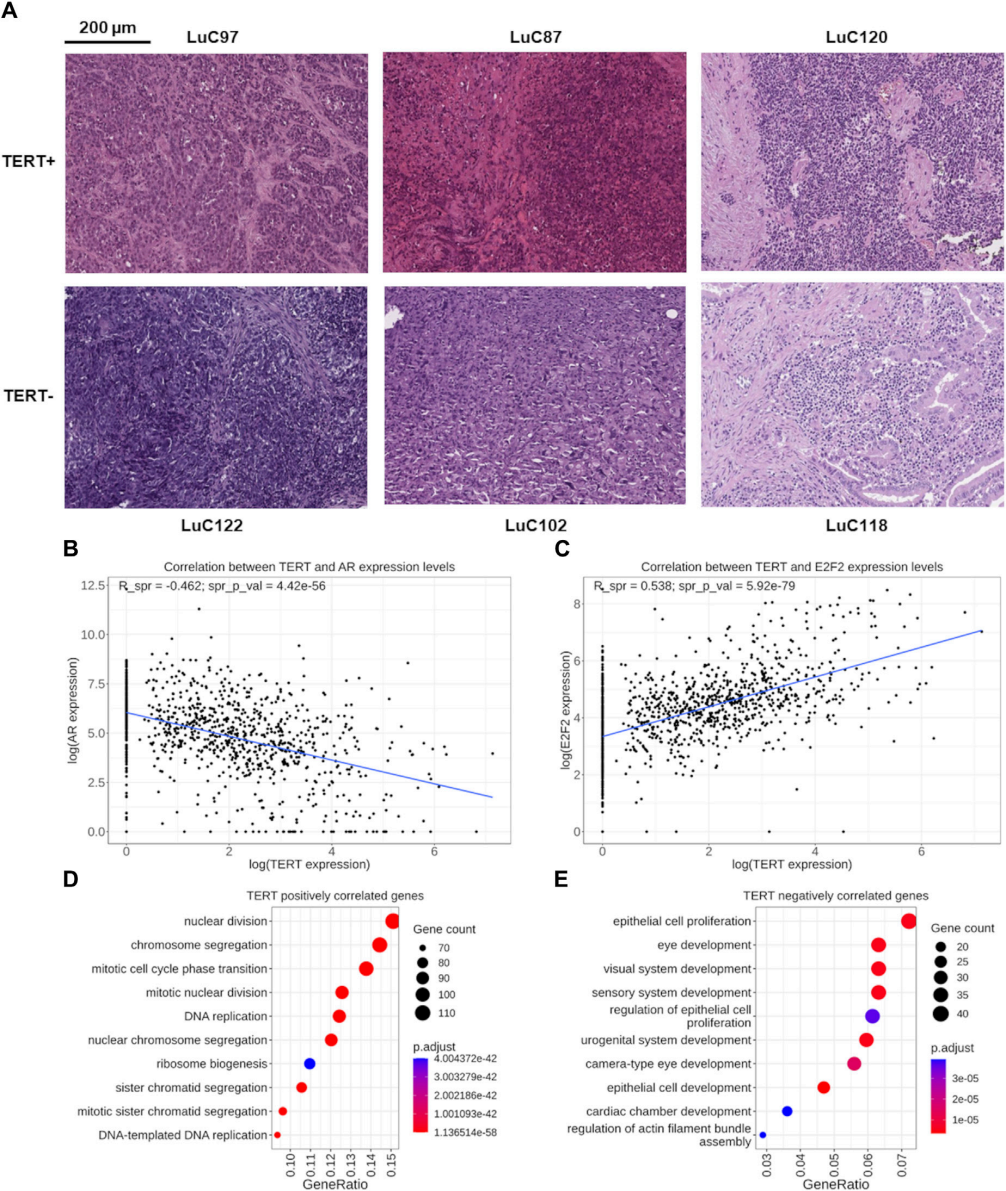
**(A)** Representative microphotographs of hematoxylin/eosin stained lung cancer biosamples. *TERT- *status was established for samples LuC122 (∼70% cancer cells), LuC102 (∼80% cancer cells), and LuC118 (∼60% cancer cells). *TERT+* status was established for samples LuC97 (∼90% cancer cells), LuC87 (∼60% cancer cells), and LuC120 (∼80% cancer cells). **(B)** Functional assessment of genes correlated with *TERT* expression. Correlation of expression levels between the *TERT* and *AR* genes. Each dot represents an individual biosample. **(C)** Correlation of expression levels between the *TERT* and *E2F2* genes. Each dot represents an individual biosample. **(D)** Gene Ontology (GO) analysis of 817 genes positively correlated with *TERT* expression. **(E)** GO analysis of 626 genes negatively correlated with *TERT* expression.

*TERT* positive or negative expression status was not correlated with the fraction of cancer cells in the tissue section evaluated by a pathologist in the biosamples under analysis (data not shown). In addition, the representative microphotographs of hematoxylin/eosin stained lung cancer biosamples show no visible differences in numbers of viable tumor cells between *TERT+* and *TERT- *tumor groups (Figure 2A).

### 3.2 Gene expression and pathway activation patterns correlating with *TERT* expression

By correlating the expression of *TERT* with other 35,125 known human genes in all 1039 cancer samples under analysis using the threshold of FDR-adjusted *p-value* less than 0.05 and Spearman correlation with absolute value more than 0.3, we identified 817 genes positively and 626 genes negatively correlated with the transcription of *TERT* (Supplementary Table S1). Among them, 103 transcription factor (TF) genes were found: 48 positively and 55 negatively correlated with *TERT* (Supplementary Table S2). Nineteen most strongly correlated TF genes with absolute value of correlation coefficient starting from 0.4 included 11 positively and 8 negatively correlated genes (Table 2).

**TABLE 2:** Transcription factor genes most strongly correlated with *TERT* expression.

Gene ID	*TERT* correlation coefficient	*p_value*	*p_adj*	Gene ID	*TERT* correlation coefficient	*p_value*	*p_adj*
*MYBL2*	0.574	3,86E-92	1,36E-87	*PURA*	−0.466	4,65E-57	2,57E-54
*E2F2*	0.538	5,92E-79	5,2E-75	*AR*	−0.462	4,42E-56	2,19E-53
*E2F1*	0.485	2,15E-62	2,47E-59	*ZNF641*	−0.422	3,17E-46	6,83E-44
*ZBED4*	0.474	2,59E-59	2,02E-56	*NFIC*	−0.412	6,33E-44	1,07E-41
*TCF3*	0.453	9,19E-54	3,63E-51	*ZNF25*	−0.409	4,02E-43	6,25E-41
*FOXM1*	0.433	9,84E-49	2,74E-46	*NFIA*	−0.408	5,57E-43	8,46E-41
*E2F8*	0.432	1,99E-48	5,31E-46	*ZFPM2*	−0.404	4,2E-42	5,9E-40
*ZNF296*	0.431	2,47E-48	6,46E-46	*CREBL2*	−0.400	3,05E-41	3,97E-39
*ZNF367*	0.418	2,95E-45	5,85E-43				
*HMGB2*	0.411	1,29E-43	2,14E-41				
*MTA2*	0.406	1,55E-42	2,27E-40				

Interestingly, among these strongly correlated 19 TF genes, for six (32%) we found previous reports in the literature that they are involved in the regulation of *TERT*. Specifically, the *AR* gene (Figure 2B) encoding the androgen receptor regulates *TERT* through chromatin binding of TFs *E2F1* and *MYC* (Dong et al., 2021). *E2F1*, *E2F2*, (Figure 2C), *E2F8* genes are representatives of the E2F family of TFs which is known as the potent inducer of *TERT* (Suenaga et al., 2006; Kumari et al., 2009; Zhang et al., 2014). The *FOXM1* (Tang et al., 2023) and *HMGB1* (Kučírek et al., 2019) genes are other known regulators of TERT stability and activity. Other strongly correlating TF genes for which we found no mentioning in the literature on their association with *TERT* were: positively correlated—*MTA2, MYBL2, TCF3, ZBED4, ZNF296, ZNF367*; negatively correlated—*CREBL2, NFIA, NFIC, PURA, ZFPM2, ZNF25, ZNF641*. Thus, the strong connection of these 13 TF genes with telomerase expression is new and may be representing new regulatory mechanisms governing transcription of *TERT*. In addition, *MYC* is a well-known regulator of *TERT* transcription, and in this study it was found statistically significantly correlated with the expression of *TERT* with a correlation coefficient of 0.33.

We then performed Gene Ontology (GO) analysis separately for the genes identified as positively or negatively correlated with *TERT* expression. We found that the pool of 817 positively correlated genes was most strongly enriched among the terms dealing with protein synthesis, ribosome biogenesis and cell division: chromatid segregation, mitosis, nuclear division, DNA replication (Figure 2D). On the other hand, the most strongly enriched GO terms among the negatively correlated 626 genes dealt mostly with the cellular terminal differentiation and organogenesis, and also with the proliferation of epithelial cells (Figure 2E).

Interestingly, the latter finding means that many genes whose expression negatively correlates with *TERT* play a role in the regulation of epithelial cell proliferation. A more careful look on these terms identified 40 genes strongly negatively correlated with *TERT*, most of which are known as positive regulators of cell proliferation (Table 3). All of these genes were statistically significantly upregulated in the *TERT*- tumors compared to the *TERT*+ group (Supplementary Figure S1). These results may indicate more complex molecular mechanisms of proliferation in *TERT- *tumors, such as alternative lengthening of telomeres (ALT) which is observed in a subset of cancers, particularly in tumors with deficiencies in telomerase activity (Zhao et al., 2019). ALT is a telomerase-independent mechanism used by cancer cells to maintain their telomeres presumably by a recombination-based pathway which could promote cell proliferation even in the case of silent telomerase genes (Zhao et al., 2019; Ferreira et al., 2020).

**TABLE 3:** Cluster of *TERT*- negatively correlated genes promoting epithelial cell proliferation.

Gene ID	*TERT* correlation coefficient	*p_value*	*p_adj*	Gene ID	*TERT* correlation coefficient	*p_value*	*p_adj*
*PURA*	−0.466	4,65E-57	2,57E-54	*MMRN2*	−0.342	6,23E-30	2,92E-28
*AR*	−0.462	4,42E-56	2,19E-53	*PGR*	−0.342	6,33E-30	2,96E-28
*VSTM4*	−0.429	9,53E-48	2,36E-45	*NRP1*	−0.339	2,01E-29	8,91E-28
*PTPRM*	−0.406	1,85E-42	2,7E-40	*FGF1*	−0.336	6,45E-29	2,7E-27
*CAV2*	−0.396	2,83E-40	3,32E-38	*KDR*	−0.336	7,67E-29	3,19E-27
*JCAD*	−0.395	3,51E-40	4,08E-38	*KLF9*	−0.330	8,2E-28	3,16E-26
*FRS2*	−0.389	6,27E-39	6,67E-37	*AGTR1*	−0.318	7,45E-26	2,42E-24
*NFIB*	−0.386	2,99E-38	2,95E-36	*AKT3*	−0.318	8,24E-26	2,66E-24
*BMPR2*	−0.386	3,01E-38	2,96E-36	*ATOH8*	−0.316	1,37E-25	4,34E-24
*ZFP36L1*	−0.384	7,61E-38	7,15E-36	*NOTCH2*	−0.316	1,59E-25	5,01E-24
*NUPR1*	−0.373	1,06E-35	8,54E-34	*YAP1*	−0.315	2,26E-25	7E-24
*SYNJ2BP*	−0.371	3,27E-35	2,45E-33	*DAB2*	−0.314	2,82E-25	8,62E-24
*TEK*	−0.363	1,16E-33	7,55E-32	*SPARC*	−0.314	3,07E-25	9,37E-24
*GLUL*	−0.360	4,66E-33	2,85E-31	*RGN*	−0.305	8,52E-24	2,23E-22
*COL8A1*	−0.354	4,49E-32	2,53E-30	*BCL2L2*	−0.305	8,97E-24	2,34E-22
*STXBP4*	−0.353	6,35E-32	3,52E-30	*NR2F2*	−0.304	1,02E-23	2,65E-22
*INTU*	−0.349	3,37E-31	1,74E-29	*FLT1*	−0.304	1,02E-23	2,66E-22
*SAV1*	−0.347	8,09E-31	4,04E-29	*NEAT1*	−0.304	1,27E-23	3,28E-22
*PRKD1*	−0.346	1,17E-30	5,8E-29	*CYP7B1*	−0.304	1,39E-23	3,58E-22
*IGFBP5*	−0.346	1,23E-30	6,04E-29	*IGFBP4*	−0.301	3,13E-23	7,74E-22

In addition, we performed an analysis also at the level of molecular pathway activation. Here the pathway activation level (PAL) metric was used to assess the correlation of *TERT* expression with the activation levels of 2929 molecular pathways. Positive PAL for pathway indicates upregulation compared with the normal tissue, negative PAL means downregulation, and zero PAL shows no changes in gene expression between tumor and normal tissues, whereas the absolute value of PAL reflects the extent of up/downregulation of a pathway in a tumor sample under analysis (Buzdin et al., 2020). Meanwhile, PAL was shown to be a promising biomarker in tumor research that is more reliable than the expression levels of individual genes (Borisov et al., 2017; Buzdin et al., 2018). In this study, PAL scores were correlated with *TERT* expression, and positive and negative links were established. Using the same criteria of FDR-adjusted *p-value* less than 0.05 and absolute value of Spearman correlation exceeding 0.3, we totally found 241 positively and 24 negatively correlated pathways with the *TERT* gene expression (Supplementary Table S3), top correlated pathways shown on Table 4.

**TABLE 4:** Top-30 molecular pathways positively correlated with expression of *TERT*.

Molecular pathway	*TERT* correlation coefficient	*p_value*	*p_adj*
reactome_Telomere_Extension_By_Telomerase_Main_Pathway	0.668	4,4E-135	1,3E-131
KEGG_Ribosome_biogenesis_in_eukaryotes_Main_Pathway	0.516	1,22E-71	1,79E-68
reactome_snRNP_Assembly_Main_Pathway	0.507	7,89E-69	7,7E-66
reactome_NoRC_negatively_regulates_rRNA_expression_Main_Pathway	0.493	1,06E-64	7,05E-62
5_aminoimidazole_ribonucleotide_biosynthesis	0.493	1,2E-64	7,05E-62
reactome_Purine_ribonucleoside_monophosphate_biosynthesis_Main_Pathway	0.487	4,09E-63	2E-60
reactome_RNA_Polymerase_I_Chain_Elongation_Main_Pathway	0.487	5,08E-63	2,13E-60
reactome_RNA_Polymerase_I_Promoter_Escape_Main_Pathway	0.486	1,12E-62	4,09E-60
reactome_Transport_of_Mature_mRNA_Derived_from_an_Intronless_Transcript_Main_Pathway	0.478	2E-60	6,52E-58
reactome_mRNA_decay_by_3_to_5_exoribonuclease_Main_Pathway	0.474	2,03E-59	5,95E-57
reactome_Rev_mediated_nuclear_export_of_HIV_RNA_Main_Pathway	0.473	3,74E-59	9,65E-57
reactome_Vpr_mediated_nuclear_import_of_PICs_Main_Pathway	0.473	3,96E-59	9,65E-57
reactome_Transport_of_the_SLBP_Dependant_Mature_mRNA_Main_Pathway	0.472	7E-59	1,58E-56
biocarta_polyadenylation_of_mRNA_Main_Pathway	0.467	2,67E-57	5,47E-55
reactome_Transport_of_the_SLBP_independent_Mature_mRNA_Main_Pathway	0.467	2,8E-57	5,47E-55
tRNA_charging	0.466	3,98E-57	7,29E-55
reactome_pre_mRNA_splicing_Main_Pathway	0.465	5,58E-57	9,61E-55
reactome_NEP_NS2_Interacts_with_the_Cellular_Export_Machinery_Main_Pathway	0.460	1,25E-55	2,04E-53
reactome_Transport_of_Mature_mRNA_derived_from_an_Intron_Containing_Transcript_Main_Pathway	0.457	7,08E-55	1,09E-52
reactome_RNA_Polymerase_I_Transcription_Termination_Main_Pathway	0.457	1,19E-54	1,74E-52
KEGG_RNA_degradation_Main_Pathway	0.456	1,73E-54	2,41E-52
reactome_Mitochondrial_tRNA_aminoacylation_Main_Pathway	0.455	2,74E-54	3,65E-52
KEGG_Spliceosome_Main_Pathway	0.455	2,96E-54	3,77E-52
reactome_Nuclear_import_of_Rev_protein_Main_Pathway	0.454	4,89E-54	5,97E-52
reactome_RNA_Polymerase_I_Transcription_Initiation_Main_Pathway	0.444	2,08E-51	2,43E-49
reactome_mRNA_Capping_Main_Pathway	0.441	1,17E-50	1,32E-48
KEGG_Basal_transcription_factors_Main_Pathway	0.440	1,88E-50	2,04E-48
reactome_Mitochondrial_transcription_initiation_Main_Pathway	0.437	9,25E-50	9,34E-48
reactome_Transport_of_Ribonucleoproteins_into_the_Host_Nucleus_Main_Pathway	0.437	9,08E-50	9,34E-48
reactome_Recycling_of_eIF2_GDP_Main_Pathway	0.436	1,55E-49	1,51E-47

Overall, the figure of top correlated pathways was remarkably similar to what was observed on the gene level. Telomerase expression was shown to strongly correlate with the pathway of telomere extension by telomerase, with the pathways of ribosomal RNA transcription and ribosome biogenesis, nuclear export and import of RNAs, ribonucleotide biosynthesis, amino acid charging of transfer RNAs, transcription and processing of messenger RNAs, and mitochondrial transcription (Table 4).

In turn, the top negatively correlated pathways dealt with the processes of cytoskeleton and external matrix remodeling, SMAD signaling, JAK degradation regulation, PTEN dependent cell cycle arrest and apoptosis, and cell proliferation through ErbB2 and ErbB3 signaling, Table 5.

**TABLE 5:** Molecular pathways negatively correlated with expression of *TERT*.

Molecular pathway	*TERT* correlation coefficient	*p_value*	*p_adj*
Regulation_of_Cytoskeleton_Remodeling_by_Activin_A	−0.406	1,84E-42	9,27E-41
NCI_E_cadherin_signaling_in_the_nascent_adherens_junction_Pathway_(regulation_of_cell_cell_adhesion)	−0.381	3,12E-37	9,62E-36
biocarta_PTEN_dependent_cell_cycle_arrest_and_apoptosis_Main_Pathway	−0.364	6,99E-34	1,76E-32
NCI_Regulation_of_nuclear_SMAD2_3_signaling_Pathway_(Pathway_degradation_of_SMAD3)	−0.356	2,54E-32	5,73E-31
biocarta_keratinocyte_differentiation_Pathway_(keratinocyte_differentiation)	−0.353	7,03E-32	1,56E-30
NCI_CDC42_signaling_events_Pathway_(cell_proliferation)	−0.345	2,04E-30	4,03E-29
NCI_Stabilization_and_expansion_of_the_E_cadherin_adherens_junction_Pathway_(actin_cable_formation)	−0.345	2,03E-30	4,03E-29
glutamine_biosynthesis	−0.345	2,42E-30	4,75E-29
reactome_TGFBR1_KD_Mutants_in_Cancer_Main_Pathway	−0.344	2,88E-30	5,54E-29
NCI_CXCR4_mediated_signaling_events_Pathway_(establishment_of_cell_polarity)	−0.342	7,07E-30	1,31E-28
reactome_SMAD2_3_MH2_Domain_Mutants_in_Cancer_Main_Pathway	−0.339	1,93E-29	3,43E-28
JAK_STAT_Pathway_JAK_Degradation	−0.332	3,32E-28	5,52E-27
NCI_Arf6_trafficking_events_Pathway_(positive_regulation_of_phagocytosis)	−0.327	2,85E-27	4,35E-26
KEGG_Cell_cycle_Main_Pathway	−0.320	3,06E-26	4,3E-25
NCI_Regulation_of_nuclear_SMAD2_3_signaling_Pathway_(muscle_cell_differentiation)	−0.318	6,49E-26	8,96E-25
Glucocorticoid_Receptor_Signaling_Pathway_Histone_Deacetylation	−0.315	2,43E-25	3,23E-24
cAMP_Pathway_Degradation_of_Cell_Cycle_Regulators	−0.314	3,29E-25	4,3E-24
NCI_ErbB2_ErbB3_signaling_events_Pathway_(cell_proliferation)	−0.309	2,11E-24	2,6E-23
reactome_The_retinoid_cycle_in_cones_daylight_vision__Main_Pathway	−0.305	9,49E-24	1,11E-22
reactome_Clathrin_derived_vesicle_budding_Main_Pathway	−0.303	1,81E-23	2,07E-22
NCI_HIF_1_alpha_transcription_factor_network_Main_Pathway	−0.302	2,26E-23	2,57E-22
biocarta_PTEN_dependent_cell_cycle_arrest_and_apoptosis_Pathway_(cell_migration)	−0.301	3,49E-23	3,92E-22
biocarta_fc_epsilon_receptor_i_signaling_in_mast_cells_Main_Pathway	−0.300	4,02E-23	4,47E-22
SMAD_Signaling_Network_Pathway	−0.300	4,19E-23	4,64E-22

**FIGURE 3:**
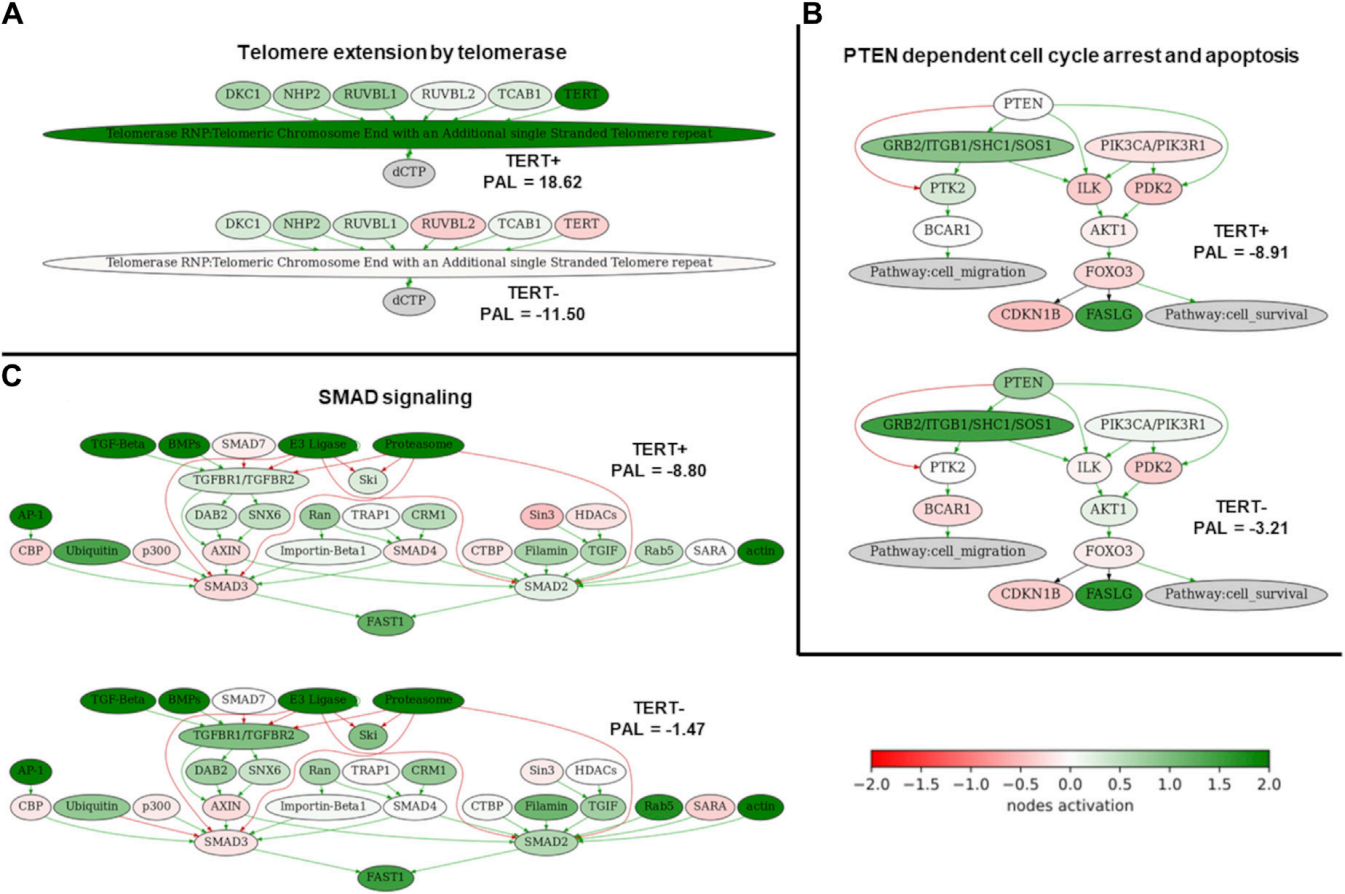
Activation profiles of three selected molecular pathways strongly correlated with the expression of *TERT*. **(A)** Telomere extension by telomerase pathway. **(B)** PTEN dependent cell cycle arrest and apoptosis pathway. **(C)** SMAD signaling pathway. Schemes for *TERT+* samples are shown on the top of each panel, for *TERT- *samples– on the bottom of each panel. Color reflects the logarithm of the case-to-normal ratio (CNR) of the pathway nodes, color scale is given (green–upregulated, red–downregulated, white–intact). Arrows show molecular interactions within a pathway: green stands for activation, red for inhibition.

Among them, the activation profiles in *TERT+* and in *TERT- *cancers can be seen in more detail for the pathways of Telomere extension by telomerase (Figure 3A), PTEN dependent cell cycle arrest and apoptosis (Figure 3B), and SMAD signaling pathway (Figure 3C). The group-averaged pathway activation charts are given relatively to the normal tissue, which was used to calculate pathway activation levels for every cancer sample under analysis. This provides a more detailed view of the differential activation of those pathway components in the *TERT+* and *TERT- *cancers.

### 3.3 Transcriptional patterns of *TERT* expression positive and negative cancers

**FIGURE 4:**
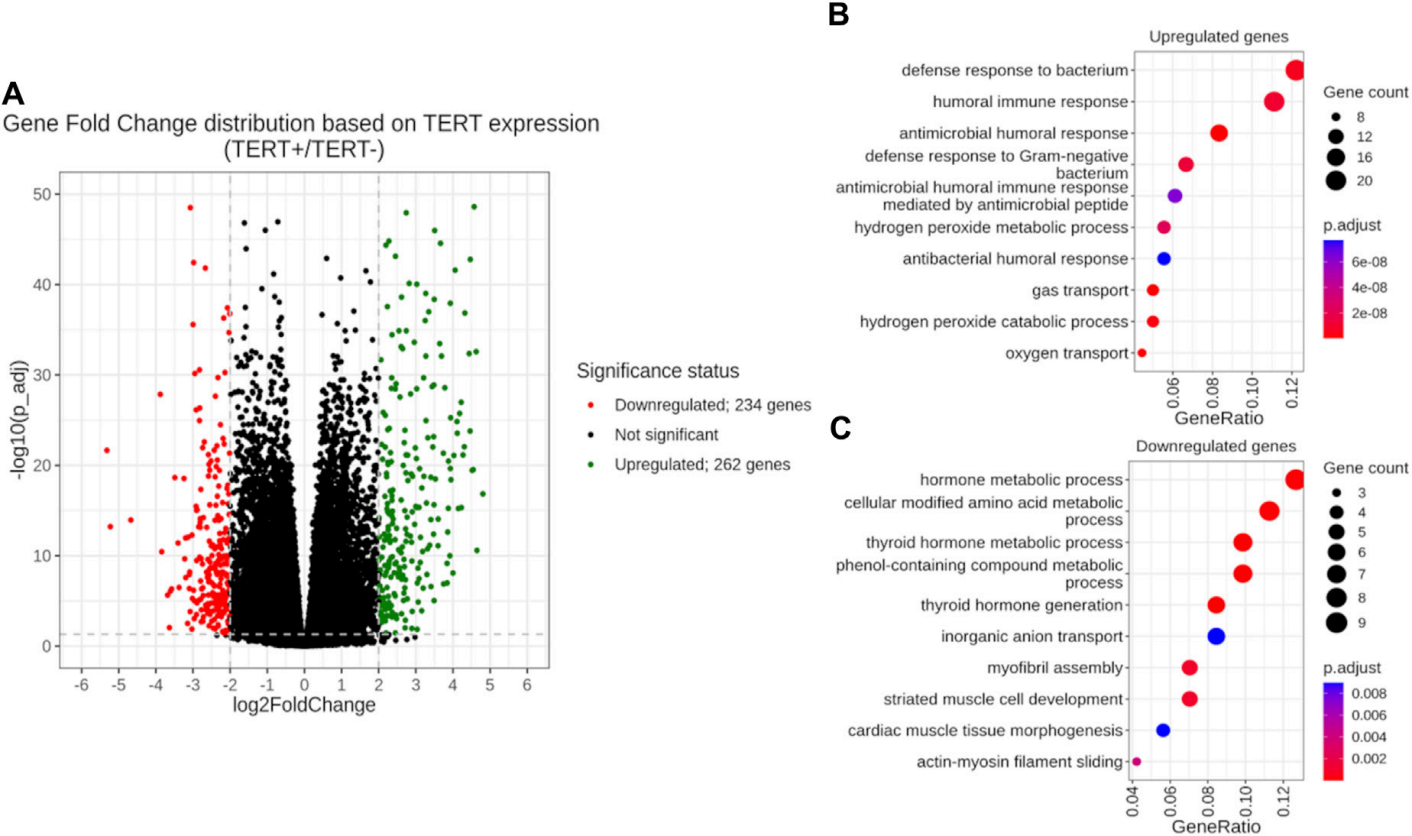
Differential gene expression analysis of *TERT+* and *TERT- *groups with a *TERT- *group taken as the reference. **(A)** Volcano plot of gene fold changes. *X*-axis, logarithm of gene fold change; *Y*-axis, negative logarithm of Benjamini-Hochberg FDR-corrected *p-value*. **(B)** GO analysis of 262 genes upregulated in *TERT +* cancers. **(C)** GO analysis of 234 genes upregulated in *TERT- *cancers.

In order to investigate differential transcriptional patterns of *TERT+* and *TERT- *cancers, we profiled differential activities of individual genes and molecular pathways where the *TERT- *group was taken as a reference. Using statistical criterion of FDR-adjusted *p-value* < 0.05 and |log2FoldChange| > 2, we identified 262 genes upregulated in *TERT*+ cancers and 234 genes upregulated in *TERT*- cancers (Figure 4A; Supplementary Table S4). At the level of molecular pathways, we found totally 1077 pathways upregulated in *TERT*+ and 898 pathways upregulated in *TERT*- cancers using non-parametric Mann Whitney U-test (Supplementary Table S5).

In *TERT*+ cancers, the group of upregulated genes was enriched in GO terms dealing with antimicrobial response, oxygen transport and hydrogen peroxide metabolism (Figure 4B). At the pathway level, in *TERT*+ cancers we observed most strongly upregulated pathways dealing with telomere extension, ribosomal RNA level and processing, ribosome biogenesis, transcription and processing of messenger RNA, nuclear transport of RNA and proteins, DNA replication and repair, and cell cycle progression (Table 6).

**TABLE 6:** Top-30 upregulated pathways in the *TERT+* group sorted by FDR-corrected *p-value* (*p_adj*).

Molecular pathway	Mean PAL (*TERT-*)	Mean PAL (*TERT+*)	*p_value*	*p_adj*
reactome_Telomere_Extension_By_Telomerase_Main_Pathway	−11,50	18,62	1,73E-63	5,05E-60
reactome_snRNP_Assembly_Main_Pathway	−3,33	11,08	8,51E-56	1,25E-52
KEGG_Ribosome_biogenesis_in_eukaryotes_Main_Pathway	−2,40	9,27	4,77E-49	4,66E-46
reactome_Transport_of_Mature_mRNA_Derived_from_an_Intronless_Transcript_Main_Pathway	−1,49	9,75	8,46E-49	6,2E-46
reactome_Transport_of_the_SLBP_Dependant_Mature_mRNA_Main_Pathway	−1,59	9,42	1,58E-48	9,27E-46
reactome_Transport_of_the_SLBP_independent_Mature_mRNA_Main_Pathway	−1,26	9,70	1,81E-47	8,26E-45
reactome_Vpr_mediated_nuclear_import_of_PICs_Main_Pathway	−1,64	9,90	1,97E-47	8,26E-45
reactome_Rev_mediated_nuclear_export_of_HIV_RNA_Main_Pathway	−0,81	10,89	4,7E-47	1,72E-44
biocarta_polyadenylation_of_mRNA_Main_Pathway	−1,54	13,30	8,48E-47	2,48E-44
reactome_NEP_NS2_Interacts_with_the_Cellular_Export_Machinery_Main_Pathway	−0,23	11,33	8,4E-47	2,48E-44
reactome_Nuclear_import_of_Rev_protein_Main_Pathway	0,15	11,74	4,81E-46	1,28E-43
reactome_Transport_of_Ribonucleoproteins_into_the_Host_Nucleus_Main_Pathway	−1,20	9,87	9,54E-44	2,33E-41
reactome_Purine_ribonucleoside_monophosphate_biosynthesis_Main_Pathway	4,73	21,24	1,99E-43	4,49E-41
reactome_CDT1_association_with_the_CDC6_ORC_origin_complex_Main_Pathway	−0,86	12,81	2,63E-42	5,51E-40
reactome_Orc1_removal_from_chromatin_Main_Pathway	−0,09	13,35	9,37E-42	1,83E-39
reactome_Fanconi_Anemia_Main_Pathway	2,55	22,55	1,15E-41	2,11E-39
reactome_APC_C_Cdh1_mediated_degradation_of_Cdc20_and_other_APC_C_Cdh1_targeted_proteins_in_late_mitosis_early_G1_Main_Pathway	0,33	13,09	2,31E-41	3,98E-39
reactome_Processing_of_Intronless_Pre_mRNAs_Main_Pathway	0,30	9,55	2,59E-41	4,22E-39
reactome_Removal_of_the_Flap_Intermediate_Main_Pathway	1,19	21,09	4,15E-41	6,4E-39
reactome_Cdc20_Phospho_APC_C_mediated_degradation_of_Cyclin_A_Main_Pathway	1,38	14,63	4,66E-41	6,83E-39
reactome_APC_C_Cdc20_mediated_degradation_of_Securin_Main_Pathway	−1,40	10,60	5,08E-41	7,08E-39
Mismatch_Repair_in_Eukaryotes_Pathway	4,22	24,53	1,6E-40	2,13E-38
reactome_Transport_of_Mature_mRNA_derived_from_an_Intron_Containing_Transcript_Main_Pathway	−3,89	4,85	2,61E-40	3,32E-38
reactome_Nuclear_Pore_Complex_NPC_Disassembly_Main_Pathway	−1,05	9,14	3,97E-40	4,85E-38
NCI_Fanconi_anemia_Pathway_(Sister_Chromatid_Exchange_Process)	5,28	24,36	4,2E-40	4,92E-38
KEGG_Fanconi_anemia_Main_Pathway	6,50	25,42	4,99E-40	5,41E-38
KEGG_Mismatch_repair_Main_Pathway	0,24	17,93	4,93E-40	5,41E-38
reactome_Separation_of_Sister_Chromatids_Main_Pathway	8,37	23,71	8,05E-40	8,42E-38
KEGG_DNA_replication_Main_Pathway	0,77	19,02	1,42E-39	1,38E-37
reactome_APC_C_Cdc20_mediated_degradation_of_Cyclin_B_Main_Pathway	4,86	20,41	1,41E-39	1,38E-37

In *TERT*- cancers, upregulated genes gave GO terms for the amino acids modifications, metabolism of hormones and phenolic compounds, transfer of inorganic anions, and assembly of myofibrils (Figure 4C). The activated pathways were related to cell cycle progression, TGF beta and SMAD signaling, JAK degradation regulation, cytoskeleton and extracellular matrix remodeling, AP1 transcription factor network, AKT signaling, PDGFR, HGF and FGFR2 signaling, epithelial to mesenchymal transition (Table 7).

**TABLE 7:** Top-30 upregulated pathways in the *TERT- *group sorted by FDR-corrected *p-value* (*p_adj*).

Molecular pathway	Mean PAL (*TERT-*)	Mean PAL (*TERT+*)	*p_value*	*p_adj*
JAK_STAT_Pathway_JAK_Degradation	4,00	−8,59	1,43E-35	5,53E-34
KEGG_Cell_cycle_Main_Pathway	−5,84	−11,99	7,77E-28	1,37E-26
Regulation_of_Cytoskeleton_Remodeling_by_Activin_A	2,63	−5,91	8,01E-27	1,36E-25
SMAD_Signaling_Network_Pathway	−1,47	−8,80	3,68E-26	5,98E-25
cAMP_Pathway_Degradation_of_Cell_Cycle_Regulators	−0,85	−7,85	1,44E-25	2,17E-24
biocarta_PTEN_dependent_cell_cycle_arrest_and_apoptosis_Main_Pathway	−3,21	−8,91	4,68E-24	6,55E-23
NCI_Regulation_of_nuclear_SMAD2_3_signaling_Pathway_(Pathway_degradation_of_SMAD3)	2,95	−4,85	8,78E-24	1,21E-22
NCI_E_cadherin_signaling_in_the_nascent_adherens_junction_Pathway_(regulation_of_cell_cell_adhesion)	2,51	−6,57	4,52E-23	6,08E-22
WNT_Pathway_Cell_Fate_Proliferation_Differentiation_Adhesion_and_Survival	0,24	−5,54	5,36E-22	6,63E-21
NCI_Integrin_linked_kinase_signaling_Pathway_(anoikis)	−10,41	−26,09	6,36E-22	7,79E-21
NCI_Regulation_of_nuclear_SMAD2_3_signaling_Pathway_(muscle_cell_differentiation)	2,04	−4,41	4,2E-21	4,8E-20
reactome_SMAD2_3_MH2_Domain_Mutants_in_Cancer_Main_Pathway	1,23	−9,97	1,14E-20	1,27E-19
NCI_ErbB2_ErbB3_signaling_events_Pathway_(cell_proliferation)	4,70	−6,26	1,27E-20	1,39E-19
ILK_Signaling_Pathway_Apoptosis	−5,70	−12,15	4,93E-20	5,25E-19
biocarta_keratinocyte_differentiation_Pathway_(keratinocyte_differentiation)	−5,54	−19,10	7,13E-20	7,51E-19
NCI_HIF_1_alpha_transcription_factor_network_Main_Pathway	−8,12	−14,96	2,22E-19	2,27E-18
reactome_TGFBR1_KD_Mutants_in_Cancer_Main_Pathway	6,50	−7,55	1,08E-18	1,07E-17
WNT_Pathway_Cytoskeletal_Rearrangement	−14,04	−23,37	3,92E-18	3,73E-17
ILK_Signaling_Pathway_MMP2_MMP9_Gene_Expression_Tissue_Invasion_via_FOS	−2,51	−14,92	6,82E-18	6,4E-17
Glucocorticoid_Receptor_Signaling_Pathway_Histone_Deacetylation	−7,27	−18,61	7,13E-18	6,65E-17
HGF_Pathway_Cell_Cycle_Progression	−9,88	−18,72	7,5E-18	6,97E-17
reactome_Smooth_Muscle_Contraction_Main_Pathway	−20,35	−37,45	9,6E-18	8,83E-17
NCI_Regulation_of_nuclear_SMAD2_3_signaling_Main_Pathway	−1,09	−6,28	1,03E-17	9,42E-17
pyridoxal_5_phosphate_salvage	8,12	−3,97	1,37E-17	1,25E-16
NCI_AP_1_transcription_factor_network_Main_Pathway	−0,47	−18,91	1,65E-17	1,49E-16
NCI_Nongenotropic_Androgen_signaling_Pathway_(cAMP_biosynthetic_process)	−11,09	−26,04	2,33E-17	2,1E-16
ATM_Pathway_G2_Mitosis_progression	−25,26	−40,11	1,74E-16	1,51E-15
reactome_TGF_beta_receptor_signaling_activates_SMADs_Main_Pathway	−1,95	−8,72	4,07E-16	3,44E-15
reactome_Clathrin_derived_vesicle_budding_Main_Pathway	−1,09	−8,64	5,44E-16	4,55E-15
NCI_PDGFR_beta_signaling_Pathway_(focal_adhesion_assembly)	−4,89	−12,70	6,47E-16	5,4E-15

Note that the majority (32/38, 84%) of DNA repair pathways was statistically significantly upregulated in *TERT*+ compared to *TERT*- cancers (Supplementary Figure S2), except for the three pathways related to ATM mediated control of G2M cell cycle checkpoint and survival and three other pathways related to p53 control of DNA integrity and survival (Supplementary Figure S2). This trend fits previous figure of incremental increase in activation of the same 32/38 pathways in the range: healthy human normal tissue — tumor adjacent pathologically normal tissue — benign tumor — malignant tumor, with the opposite regulation for the six pathways related to ATM and p53 replication control (Vladimirova et al., 2021; Sorokin et al., 2023; Zolotovskaia et al., 2023).

Thus, we detected an overally differential trend where *TERT*+ cancers showed the increased transcription and translation, and DNA replication backed by enhanced DNA repair with disrupted quality checkpoints. In contrast, *TERT*- cancers showed the activation of peculiar set of positive cell cycle promoting genes and strong epithelial-to-mesenchymal transition marks (Table 7).

### 3.4 Transcriptional patterns associated with mutations in *TERT* promoter

**FIGURE 5:**
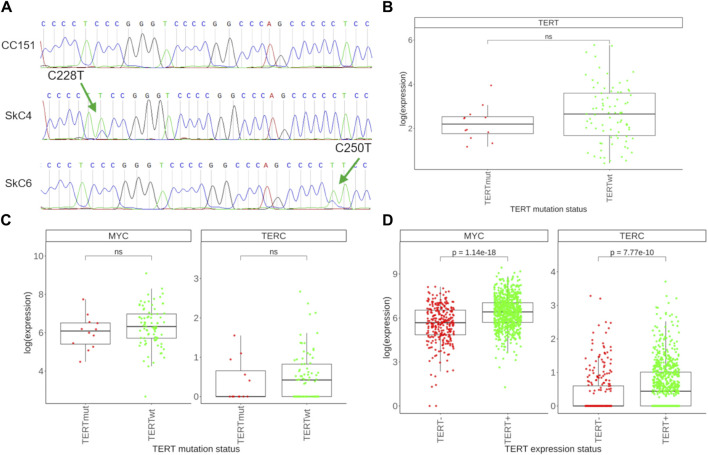
Assessment of *TERT* promoter mutations and associated transcriptional features. **(A)** Sanger-sequenced profile of the *TERT* gene promoter region spanning the putative *C228T* and *C250T* mutation sites. Examples are shown of no mutations found (top), of *C228T* mutation (center), and of *C250T* mutation identified (bottom). Green arrows indicate the position of the mutation sites. Tumor sample IDs are shown left to the respective tracks. **(B)** Expression of the *TERT* gene in 12 *TERTmut* and 81 *TERTwt* tumors. **(C)** Expression of the *MYC* and *TERC* genes in *TERTmut* and *TERTwt* tumors. **(D)** Expression of the *MYC* and *TERC* genes in 278 *TERT*- tumors and 761 *TERT*+ tumors.

We then tried to characterize transcriptional patterns that could be associated with *TERT* promoter mutation status in the group of *TERT*- expressing (*TERT*+) cancers. To this end we collected where possible fragments of remaining biomaterials available after extraction of RNA for sequencing and proposed to the patients or their legal representatives to sign a new informed agreement form to participate in the investigation of molecular features associated with *C228T/A* and *C250T/A* mutations in *TERT* promoter. Both conditions were met for 114 patient cases, and our further mutation screening identified among them 101 cases with wild-type *TERT* promoter and 13 cases with the presence of either mutation (Table 1). *TERT* promoter mutations were found in 5 glioblastomas, 3 melanomas, 2 skin carcinomas, 2 urothelial carcinomas, and in one thyroid cancer sample (Figure 5A). For further analysis, we took only the samples showing detectable *TERT* expression, and selected totally 81 *TERTwt* and 12 *TERTmut* profiles. Since the sampling of tumor samples with known status of *TERT* promoter mutations was limited, our findings of comparing the *TERTwt* and *TERTmut* groups should be considered as the pilot study results that need to be validated on greater cohorts in the future.

We first compared expression of *TERT* in selected *TERTmut* and *TERTwt* tumors and found lack of detectable difference among the two groups (Figure 5B). This was also the case for the expression of *TERC*, a gene for the RNA component of telomerase (Figure 5C). We also found no difference for the expression levels of *MYC*, a known potent inducer of *TERT* in normal and malignant tissues (Figure 5C). At the same time, both *TERC* and *MYC* were found significantly upregulated in the group of *TERT*+ cancers compared to the *TERT*- group (Figure 5D). Thus, our results indicate that mutations in *TERT* promoter do not result in an overexpression of telomerase components compared to other *TERT*+ tumors.

**FIGURE 6:**
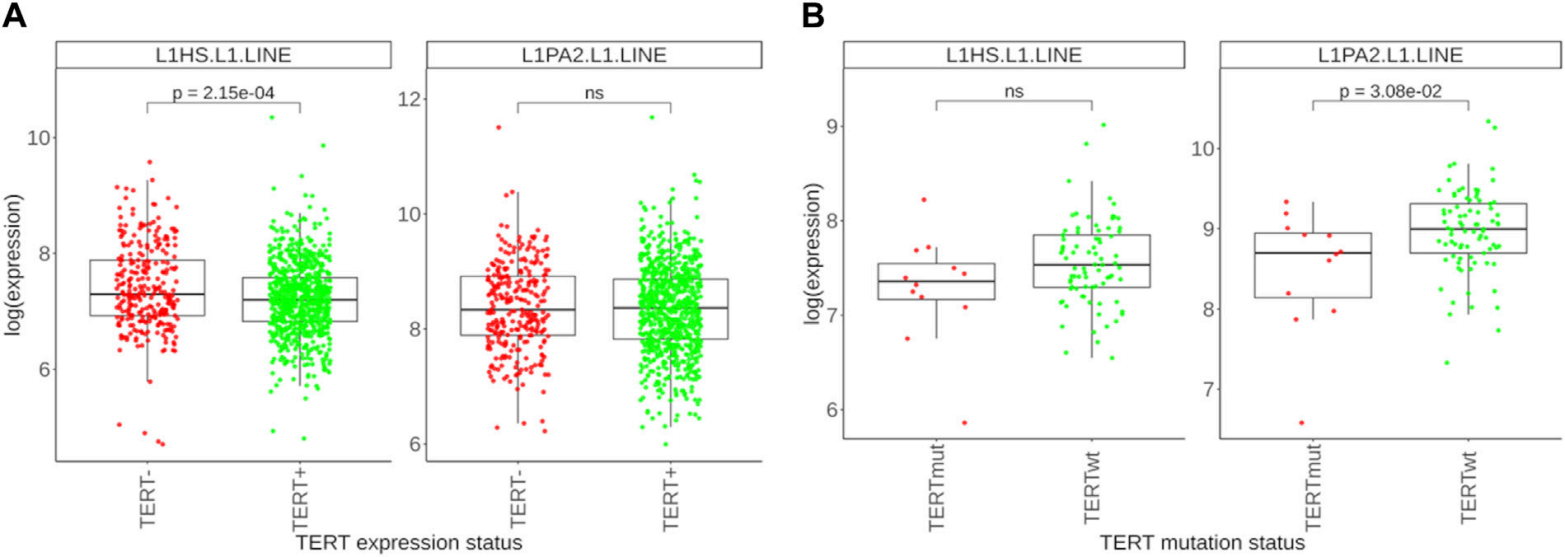
Differential expression profile of L1 retrotransposon subfamilies. **(A)** Expression levels of L1HS and L1PA2 retrotransposons in 278 *TERT*- tumors and 761 *TERT*+ tumors. **(B)** Expression levels of L1HS and L1PA2 retrotransposons in 12 *TERTmut* and 81 *TERTwt* tumors.

In addition to the multiple important non-canonical roles of *TERT* including regulation of apoptosis, maintenance of the cellular redox homeostasis, the main function of active telomerase is adding specific DNA sequence monomers to chromosome ends thus preventing their pathologic shortening following a series of cell division cycles. However, in some organisms this role is played by the LINE retrotransposons which insert their DNA copies in the chromosome termini (Shpiz et al., 2007). In humans, L1 family of LINE retrotransposons occupies nearly 20% of the DNA (Suntsova and Buzdin, 2020) and is fully active thus generating new inserts through the genome (Terry and Devine, 2019). L1 retrotransposons can also mobilize other elements and may cause many kinds of DNA rearrangements (Schumann et al., 2010). Importantly, L1 retrotransposition may deal with the function of rescuing the chromosome ends in cancer independently of telomerase (Aschacher et al., 2020), as shown experimentally for glioblastoma. Furthermore, L1s are known to be transcriptionally reactivated in human cancers (Protasova et al., 2021; Mathavarajah and Dellaire, 2024). In the human genome, two subfamilies of L1 (L1HS and L1PA2) were reported to be able to transpose (Buzdin et al., 2003). Thus, we compared expression levels of these two L1 subfamilies in our experimental set of *TERT*-, *TERT*+, *TERTmut* and *TERTwt* tumors (Figure 6). Firstly, we detected a higher expression of L1 retrotransposons in *TERT*- compared to *TERT*+ tumors (statistically significant for L1HS subfamily, Figure 6A). Secondly, there was a lower expression of L1 elements in *TERTmut* compared to *TERTwt TERT*+ tumors (statistically significant for L1PA2; a trend is seen also for the L1HS elements, Figure 6B). Thus, we found that the factors known to be associated with telomerase reactivation (increased expression, activating promoter mutations) are also associated with the decrease of active L1 subfamilies expression, and *vice versa*. However, the ALT pathway could also be associated with a higher likelihood of genomic rearrangements, including the activation of L1 elements, which potentially causes genomic instability and contributes to tumorigenesis. Conversely, in *TERT+* tumors with active telomerase, where telomere maintenance primarily occurs through the *TERT* function, the L1 activity is instead reduced (Aschacher et al., 2020).

To summarize, in this study we experimentally investigated gene expression patterns associated with the status of *TERT* transcription on a pan-cancer level. We found a bimodal distribution of *TERT* expression where ∼27% of cancer samples did not express *TERT*. In turn, expression of *TERT* was strongly correlated with 1443 human genes and 265 molecular pathways. We also found differentially activated 496 genes and 1975 molecular pathways for the comparison of *TERT*- positive and negative cancers, which may reflect greater physiological impact of changes in the molecular pathways compared to the individual genes. Our pilot assay showed that the *TERT+* group there was ∼13% of cancers with mutated *TERT* promoter, where the presence of promoter mutations was not associated with a greater level of *TERT* expression. Taken together, this evidences parallel mechanisms leading to the *TERT* gene transcriptional activation, and also parallel mechanisms of proliferation and survival in *TERT*- positive and negative cancers. Finally, we detected a decreased expression of L1 retrotransposons in the *TERT*+ group, and further decreased L1 expression in promoter mutated *TERT*+ cancers.

## 4 Discussion

In over 80% of tumors, the increased *TERT* gene expression promotes abnormal telomerase activation and upregulated telomere maintenance mechanism (Lee et al., 2016; Dratwa et al., 2020; Liu et al., 2024). In the context of cancer development, *TERT* serves as a critical factor by contributing to the maintenance of chromosomal stability through its role in preserving telomere length (Colebatch et al., 2019). In cancer cells, it serves as a constraining element in the assembly of the telomerase complex (Dratwa et al., 2020).

However, the important roles of *TERT* in vital non-canonical processes other than telomerase activity should be also considered. For example, *TERT* can act as a modulator in NF-kB and Wnt/β-catenin signaling pathways, regulate gene expressions, proliferation, apoptosis, cell cycle, chromosomal rearrangements, epigenetic modifications (Li and Tergaonkar, 2014; Dratwa et al., 2020; Liu et al., 2024). At the same time, in tumor cells with deficiencies in telomerase activity, telomere elongation can occur due to the telomerase-independent ALT pathway (Zhao et al., 2019). Thus, alterations in *TERT* expression can lead to disruption of the above molecular mechanisms, which may have a role in tumorigenesis.

In this study, the pathway and differential gene analysis revealed the complex influence of *TERT* expression on multiple aspects of cancer cell physiology. For example, *TERT* expression was positively associated with many pathways mediating enhanced transcription, ribosome assembly, translation, replication, DNA repair, and cell division (Figure 2; Table 4). The upregulation of the ribosome biogenesis pathway could be due to various factors related to telomerase activity. One interpretation of this pathway being prominently upregulated in the context of telomerase reactivation could be related to the association of *TERC* with dyskerin, a component of the H/ACA ribonucleoprotein complex involved in ribosome biogenesis (Montanaro et al., 2008). Also, the protein encoded by the *NAT10* gene is a part of the “ribosome biogenesis” GO term and participates in the acetylation of telomeric proteins (Lv et al., 2003; Fu and Collins, 2007). In general, the upregulation of ribosome biogenesis may reflect the increased need for ribosomes to facilitate the translation of mRNA into proteins during forced division of tumor cells (Gonzalez et al., 2014). Little or no expression of *TERT* was connected with the activation of pathways related to epithelial-to-mesenchymal transition (Figure 2; Table 5), and with a number of cell cycle and survival promoting gene products (Table 3). On Supplementary Figure S3 we schematized major cellular processes differentially regulated in TERT+/− tumors. However, the results obtained obviously reflect the complex genetic heterogeneity of the analyzed tumors in pan-cancer and the lack of possibility to investigate effects of *TERT+/TERT- *expression status in the same genetic background, as it could be implemented on experimental *in vivo* models.

Many tumors, such as gliomas, melanomas, and urothelial carcinomas show a high frequency of *TERT* promoter mutations, and *TERT* with mutations in its promoter can be highly expressed that can lead to abnormally enhanced telomerase activity (Borah et al., 2015; Heidenreich et al., 2015; Lee et al., 2016; Shaughnessy et al., 2020; Guo et al., 2022). For example, the increased *TERT* expression in mutant melanoma cell lines was associated with the MAPK signaling pathway, which constitutively activates the transcription factor ETS binding to the mutant *TERT* promoter and increasing its transcription (Vallarelli et al., 2016). However, several reports regarding thyroid cancer, bladder cancer, melanoma, and breast cancer indicate no connection between *TERT* promoter mutations and the extent of this gene expression (Allory et al., 2014; Pestana et al., 2020; Salgado et al., 2020). Our analysis on a pan-cancer level also revealed no statistically significant differences in the *TERT* gene expression between the groups of *TERTmut* and *TERTwt* tumors. These results may be associated with parallel *TERT* transcriptional regulation pathways, where methylation of the *TERT* promoter is one of the well-known regulatory mechanisms. For example, *TERT* promoter methylation is counterintuitively correlated with chromatin accessibility and *TERT* expression (Salgado et al., 2020; Liu et al., 2023). Also, the formation of G-quadruplexes in the G-rich region of the promoter has a major influence on the expression of *TERT*, and mutations in the G-rich region of the promoter may lead to disruption of their structure which in turn strongly affects transcription factor binding and enhances *TERT* expression (Gu et al., 2022; Pavlova et al., 2022).

We also found that the expression of active human retrotransposon family L1 is increased in the *TERT*- deficient group of cancers (Figure 6). Interestingly, connection between the transcription of L1 and *TERT* was previously reported for cancer cells (Aschacher et al., 2016). It has to be investigated in the future whether this may be somehow related to the hypothetical function of telomere extension by the L1 reverse transcriptase, or does it simply represent occasional transcriptional interplay between the processes. It is important to note that L1 elements are able to retrotranspose into telomere regions and may be a part of the alternative lengthening of telomeres (ALT) mechanism that operates independently of the telomerase and, therefore, potentially contributes to the elongation of telomeres in ALT-positive cells (Aschacher et al., 2020).

Finally, our analysis revealed 1443 genes whose expression is strongly correlated with the transcription of *TERT*. Among them, 17 genes represent molecular targets of cancer therapeutics, where 9 genes are positively and 8 negatively correlated with the transcription of *TERT* (Table 8).

**TABLE 8:** Cancer drug target genes which are positively and negatively correlated with *TERT* expression.

Drug target gene ID	*TERT* correlation coefficient	Up/downregulated in the *TERT+* group	Cancer drug generic names
*PLK1*	0.419	Up	Rigosertib
*PARP1*	0.391	Up	Niraparib; Olaparib; Rucaparib; Talazoparib; Veliparib
*CDK1*	0.361	Up	Flavopiridol (Alvocidib)
*TUBA4A*	0.337	Up	Ado-Trastuzumab Emtansine; Brentuximab vedotin; Enfortumab vedotin; Vinblastine; Vincristine; Vindesine; Vinorelbine
*MAP2K2*	0.334	Up	Binimetinib (MEK162); Selumetinib; Trametinib (Mekinst)
*PSMB10*	0.326	Up	Carfilzomib
*IDH2*	0.324	Up	Enasidenib
*TUBB4B*	0.308	Up	Cabazitaxel; Docetaxel; Eribulin; Ixabepilone; Paclitaxel
*TUBA1B*	0.303	Up	Ado-Trastuzumab Emtansine; Brentuximab vedotin; Enfortumab vedotin; Vinblastine; Vincristine; Vindesine; Vinorelbine
*AR*	−0.462	Down	Abiraterone; Apalutamide, ARN-509; Bicalutamide; Buserelin; Cyproterone acetate; Degarelix; Dienogest; Enzalutamide; Fluoxymesterone; Flutamide; Goserelin; Histrelin; Leuprolide; Levonorgestrel; Medroxyprogesterone acetate (MPA); Methyltestosterone; Nilutamide; Triptorelin
*NTRK2*	−0.372	Down	Entrectinib; Larotrectinib
*TEK*	−0.363	Down	Foretinib; Regorafenib
*PGR*	−0.342	Down	Dienogest; Levonorgestrel; Medroxyprogesterone acetate (MPA); Megestrol
*KDR*	−0.336	Down	Axitinib; Cabozantinib; Dovitinib; Foretinib; Lenvatinib; Midostaurin; Nintedanib (BIBF 1120); Pazopanib; Ramucirumab (Cyramza); Regorafenib; Sorafenib; Sunitinib; Tivozanib; Vandetanib
*AKT3*	−0.318	Down	Perifosine
*RARB*	−0.313	Down	Acitretin; Alitretinoin
*FLT1*	−0.304	Down	Axitinib; Dovitinib; Lenvatinib; Nintedanib (BIBF 1120); Pazopanib; Regorafenib; Sorafenib; Sunitinib; Tivozanib

**FIGURE 7:**
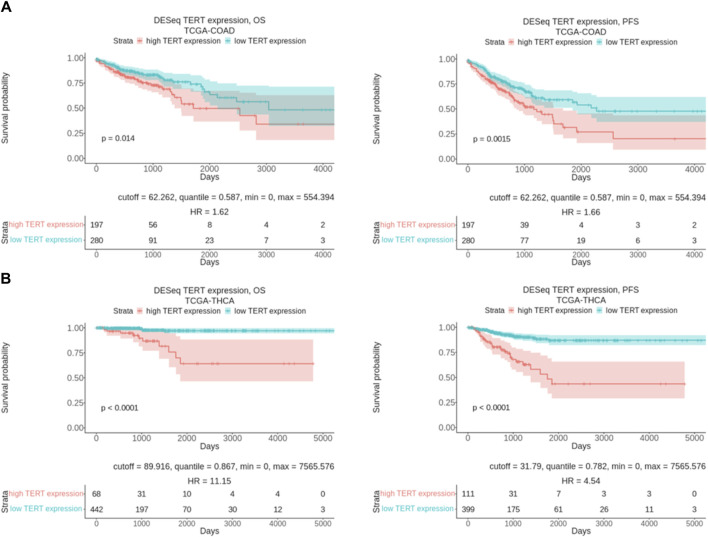
Overall and progression free survival analysis results in relation with *TERT* expression level. **(A)** Kaplan-Meier plots built for colorectal cancer patients from TCGA database (TCGA-COAD). **(B)** Kaplan-Meier plots built for thyroid cancer patients from TCGA database (TCGA-THCA). OS means overall survival and PFS means progression free survival.

Thus, our findings may shed light on the differential sensitivity of tumors on targeted therapies depending on the *TERT* expression status. *TERT* expression level is known to be a prognostic survival biomarker in many cancers. In agreement with this, our pilot assessment of overall survival (OS) and progression-free survival (PFS) data from the TCGA project for colorectal cancer (TCGA-COAD) and thyroid cancer (TCGA-THCA) revealed a strong association of lower expression of *TERT* with a favorable outcome (Figure 7). Thus, it has to be investigated in the future whether *TERT* expression typing of cancers may serve to personalize existing modes of therapy or even develop new approaches for the differential treatment of patients.

For the first time, in this comprehensive study of a 1039-patient cohort we have tried to catalogue these and other molecular processes connected with the transcription of the *TERT* gene in cancers. We hope that the results communicated here may be useful to those interested in different ways of proliferation and survival of human cancer cells or working in the domain of advanced molecular diagnostics.

## 5 Conclusion

Here we experimentally assessed the gene expression patterns associated with the status of *TERT* transcription in 1039 human cancer samples of 27 tumor types investigated by RNA sequencing. We showed a bimodal distribution of *TERT* expression where ∼27% of cancer samples did not express *TERT*, whereas the rest showed a bell-shaped distribution. Expression of *TERT* was strongly correlated with 1443 human genes including 103 encoding transcriptional factor proteins. Comparison of *TERT*- positive and negative cancers showed the differential activation of 496 genes and 1975 molecular pathways. Among them, 32/38 (84%) of DNA repair pathways were hyperactivated in *TERT+* cancers which was also connected with accelerated replication, transcription, translation, and cell cycle progression. In contrast, the level of 40 positive cell cycle regulator proteins was specific for the *TERT*- group which suggests different proliferation strategies for both groups of cancer. Our pilot study showed that the *TERT+* group had ∼13% of cancers with mutated *TERT* promoter (mutations *C228T*, *C250T*, or both). However, the presence of promoter mutations was not associated with a greater level of *TERT* expression compared with other *TERT*+ cancers. This evidences parallel mechanisms leading to the *TERT* gene transcriptional activation. In addition, we detected a decreased expression of L1 retrotransposons in the *TERT*+ group, and further decreased L1 expression in promoter mutated *TERT*+ cancers. *TERT* expression was correlated with 17 target genes for cancer therapy which may relate to differential survival pattern of *TERT*- positive and negative cancers.

## Data Availability

The data analyzed in this study is subject to the following licenses/restrictions: Patient raw genetic and gene expression data obtained in this study cannot be submitted to public databases due to patient written informed consent formulation. However, normalized gene expression and TERT promoter mutation data are available upon the request to the authors. Requests to access these datasets should be directed to AB, buzdin@oncobox.com.
