# Supplementary material for: Pan-cancer experimental characteristic of human transcriptional patterns connected with telomerase reverse transcriptase (TERT) gene expression status
Source: Front Genet. 2024 May 27;15:1401100. doi: 10.3389/fgene.2024.1401100 (PMC11163056; doi:10.3389/fgene.2024.1401100)
Supplement: Supplementary file 1 [file DataSheet1.ZIP › Supplementary_Figure1_rev.pdf]

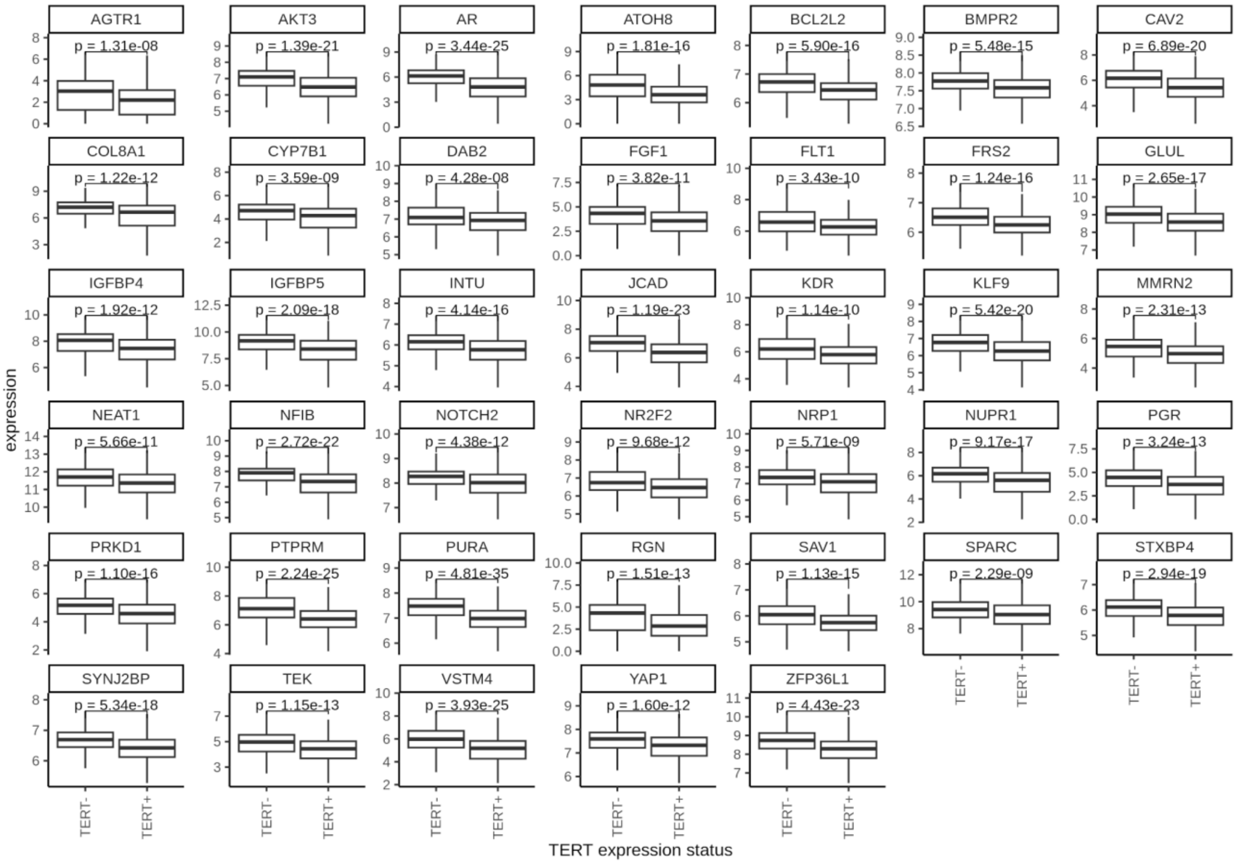

Supplementary Figure S1. Differential expression analysis in *TERT+* and *TERT-* groups of tumors for 40 genes negatively correlated with *TERT* expression and involved in the positive regulation of epithelial cell proliferation. X-axis, *TERT* expression status; Y-axis, natural logarithm of gene expression. For statistical estimates of differential gene expression between *TERT+* and *TERT-* samples, the non-parametric Mann–Whitney U-test was used.
