# Supplementary material for: Pan-cancer experimental characteristic of human transcriptional patterns connected with telomerase reverse transcriptase (TERT) gene expression status
Source: Front Genet. 2024 May 27;15:1401100. doi: 10.3389/fgene.2024.1401100 (PMC11163056; doi:10.3389/fgene.2024.1401100)
Supplement: Supplementary file 1 [file DataSheet1.ZIP › Supplementary_Figure2_rev.pdf]

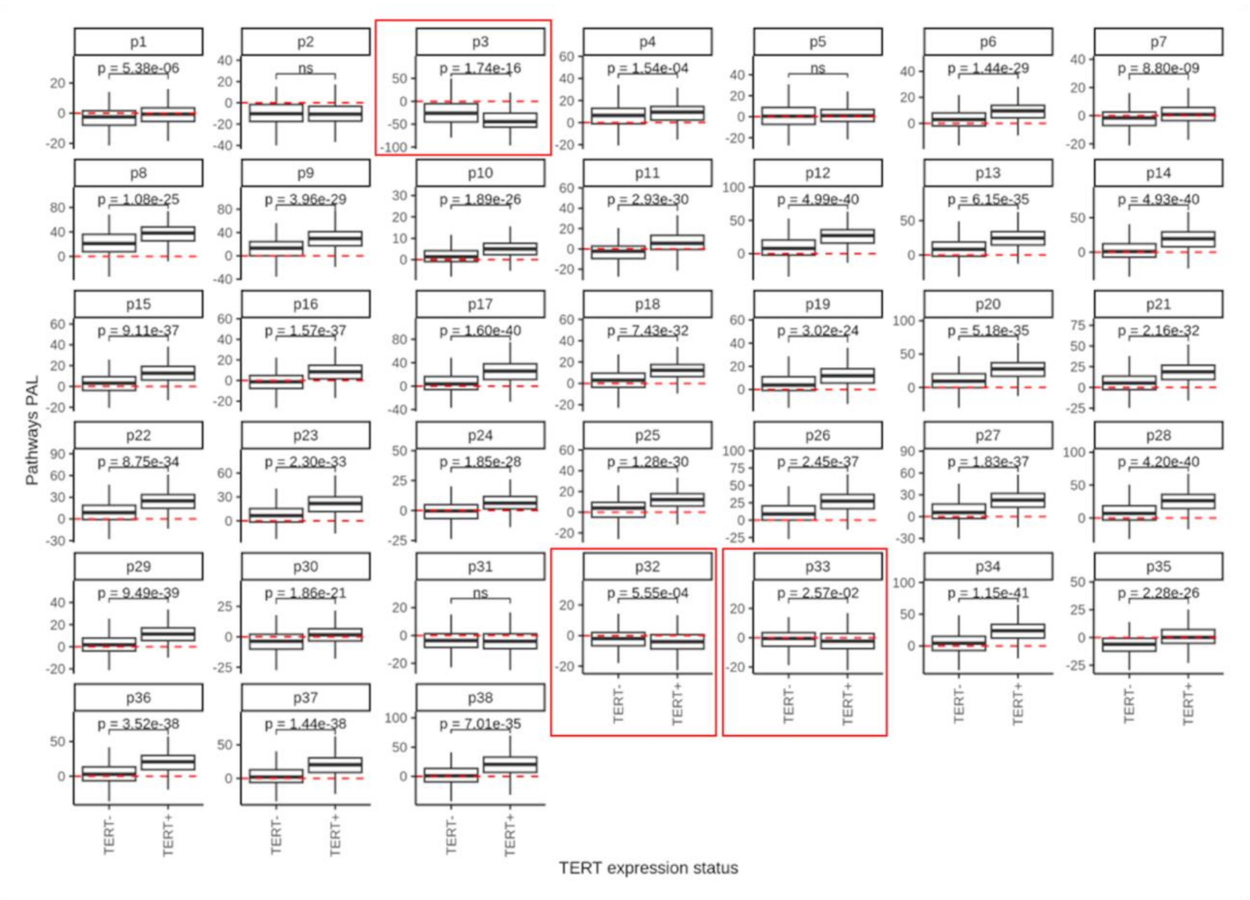

Supplementary Figure S2. Comparison of DNA repair pathway activation profiles in *TERT+* and *TERT-* cancers. Red frames indicate pathways upregulated in *TERT-* cancers. The following molecular pathways were assessed: p1 - ATM Pathway; p2 - ATM Pathway Cell Survival; p3 - ATM Pathway G2/Mitosis progression; p4 - biocarta atm signaling Main Pathway; p5 - biocarta cell cycle g2 m checkpoint Main Pathway; p6 - BRCA1 Pathway; p7 - BRCA1 Pathway Chromatin Remodeling; p8 - BRCA1 Pathway Homologous Recombination Repair; p9 - BRCA1 Pathway Mismatch Repair; p10 - DNA Repair Mechanisms Pathway; p11 - KEGG Base excision repair Main Pathway; p12 - KEGG Fanconi anemia Main Pathway; p13 - KEGG Homologous recombination Main Pathway; p14 - KEGG Mismatch repair Main Pathway; p15 - KEGG Non homologous end joining Main Pathway; p16 - KEGG Nucleotide excision repair Main Pathway; p17 - Mismatch Repair in Eukaryotes Pathway; p18 - NCI ATM Main Pathway; p19 - NCI ATM Pathway (G1 S transition checkpoint); p20 - NCI ATR signaling Main Pathway; p21 - NCI ATR signaling Pathway (Pathway negative regulation of transcription during mitosis via CHEK1); p22 - NCI ATR signaling Pathway (regulation of double strand break repair via homologous recombination); p23 - NCI ATR signaling Pathway (response to G2 M transition DNA damage checkpoint signal); p24 - NCI DNA PK pathway in nonhomologous end joining Pathway (double strand break repair via nonhomologous end joining); p25 - NCI DNA PK pathway in nonhomologous end joining Pathway (V D J recombination); p26 - NCI Fanconi anemia Main Pathway; p27 - NCI Fanconi anemia Pathway (regulation of double strand break repair via homologous recombination); p28 - NCI Fanconi anemia Pathway (Sister Chromatid Exchange Process); p29 - NHEJ mechanisms of DSBs repair effect; p30 - Nucleotide excision repair effect; p31 - p53 Signaling Pathway; p32 - p53 Signaling Pathway DNA Repair; p33 - p53 Signaling Pathway Gene Expression DNA Replication and Repair via TP53; p34 - reactome Fanconi Anemia Main Pathway; p35 - reactome Formation of transcription coupled NER TC NER repair complex Main Pathway; p36 - reactome Mismatch repair MMR directed by MSH2 MSH3 MutSbeta Main Pathway; p37 - reactome Mismatch repair MMR directed by MSH2 MSH6 MutSalpha Main

Pathway; p38 - reactome Repair synthesis for gap filling by DNA polymerase in TC NER Main Pathway.
