# Supplementary material for: Pan-cancer experimental characteristic of human transcriptional patterns connected with telomerase reverse transcriptase (TERT) gene expression status
Source: Front Genet. 2024 May 27;15:1401100. doi: 10.3389/fgene.2024.1401100 (PMC11163056; doi:10.3389/fgene.2024.1401100)
Supplement: Supplementary file 1 [file Supplementary_Figure3_rev.pdf › Supplementary_Figure3_rev.pdf]

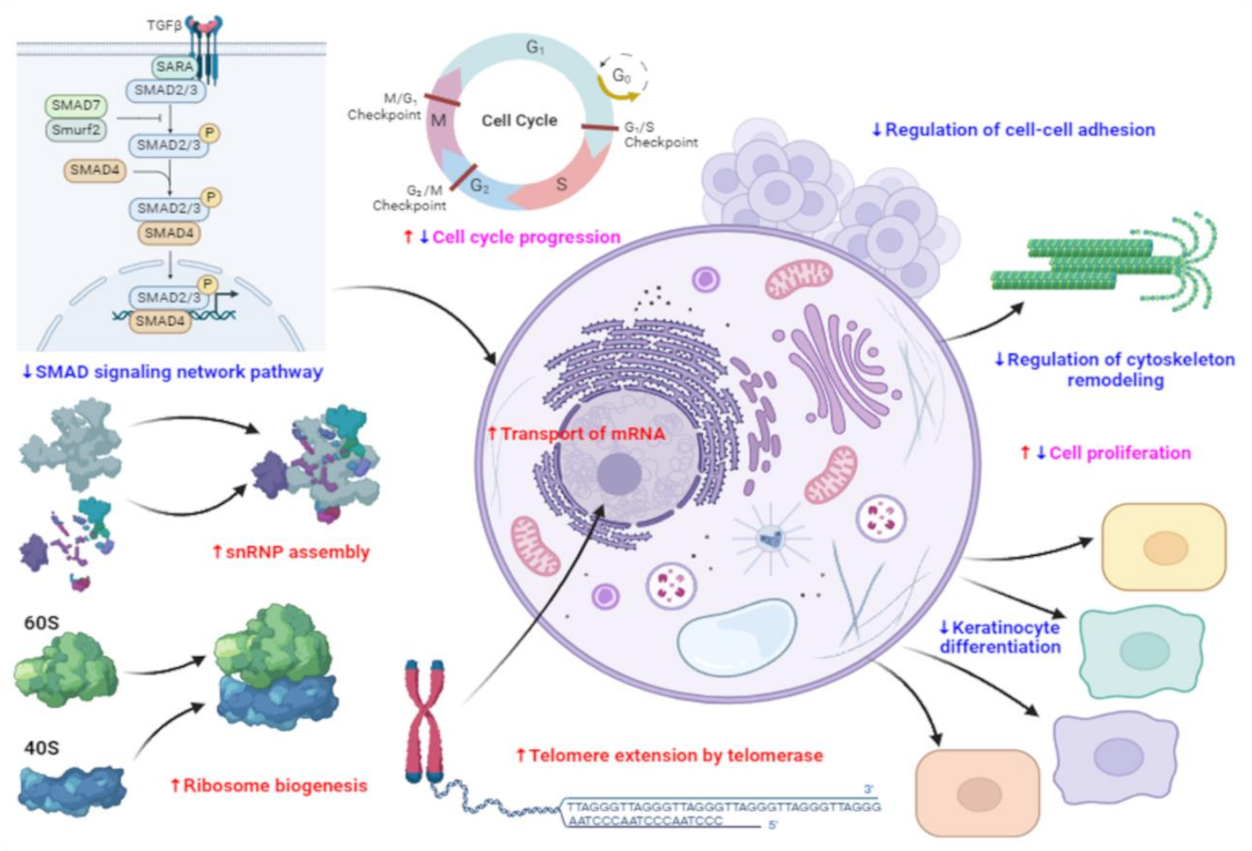

Supplementary Figure S3. Schematic representation of cellular processes differentially activated in *TERT+* (arrow up) and in *TERT-* tumors (arrow down). Red text denotes the processes positively correlated, blue – negatively correlated with the expression of *TERT*; magenta indicates the processes which include both positively and negatively correlated pathways. Visualized with BioRender.com.
